# Oxidative Stress and Apoptosis Inhibition Mitigate Static Cold Storage-Induced Injury in Liver Sinusoidal Endothelial Cells

**DOI:** 10.3390/cells15151334

**Published:** 2026-07-25

**Authors:** Bradley W. Ellis, Huyun Chen, Mohammadreza Mojoudi, Alban Longchamp, Heidi Yeh, Martin L. Yarmush, Mehmet Toner, Korkut Uygun, Basak E. Uygun

**Affiliations:** 1 Center for Engineering in Medicine and Surgery, Massachusetts General Hospital, Harvard Medical School, Boston, MA 02114, USA; 2 Shriners Children’s Boston, Boston, MA 02114, USA; 3 Center for Transplantation Sciences, Massachusetts General Hospital, Harvard Medical School, Boston, MA 02114, USA; 4 Department of Biomedical Engineering, Rutgers University, Piscataway, NJ 08854, USA

**Keywords:** liver sinusoidal endothelial cells, endothelial preservation, liver transplant, ischemia reperfusion injury, apoptosis inhibition, oxidative stress inhibition

## Abstract

**Highlights:**

**What are the main findings?**

- Liver sinusoidal endothelial cells (LSECs) exhibit a distinct static cold storage-induced preservation injury profile compared with other hepatic cells.
- Inhibition of apoptosis and oxidative stress significantly improve LSEC viability and functionality following preservation injury.

**What are the implications of the main findings?**

- Apoptosis and oxidative stress inhibition represent promising therapeutic approaches for conventional and emerging liver preservation strategies.
- Results highlight the importance of understanding LSEC-specific injury mechanisms to provide endothelial-targeted preservation strategies.

**Abstract:**

Donor liver scarcity is exacerbated by preservation-related injury, particularly ischemia-reperfusion injury (IRI), which reduces organ utilization and increases discard rates. Liver sinusoidal endothelial cells (LSECs) play a critical role in mediating IRI, yet their response to static cold storage (SCS) remains poorly understood. Here, we investigate the impact of SCS on isolated rat LSECs. Isolated rat hepatocytes, stellate cells, Kupffer cells, and LSECs were subjected to up to 3 days of SCS followed by up to 2 days of recovery, with LSECs also receiving apoptosis and/or oxidative stress inhibition. Afterwards, changes in survivability, functionality, and morphology were measured. Additionally, changes in gene, cytokine, and chemokine expression were also measured. We found that SCS reduced cell viability by approximately 40%, accompanied by a 60% reduction in metabolic activity and ATP levels, indicating substantial impairment in cellular energetics. SCS also doubled reactive oxygen species (ROS) production and upregulated oxidative stress and apoptosis-related genes, leading to functional decline in LSECs. Importantly, combined inhibition of apoptosis and oxidative stress improved LSEC viability by 20% and metabolic activity and ATP levels by 30% and 40%, respectively, and reduced ROS production by 50%. These findings highlight LSEC vulnerability to preservation injury and the importance of understanding LSEC-specific injury mechanisms to provide a foundation for the development of endothelial-targeted preservation strategies to significantly improve liver transplantation outcomes, subsequently increasing access to this life-saving treatment.

## 1. Introduction

Orthotopic liver transplantation remains the only definitive long-term treatment for end-stage liver disease [1,2,3,4]. Although more than 30,000 liver transplants are performed annually, this number meets less than 10% of the global demand [5]. Despite high post-transplant survival rates, the limited availability of viable donor organs results in thousands of patients dying each year while awaiting transplantation [2,6,7,8]. This shortage is compounded by the short permissible preservation window, which restricts the ability to transport organs across wider geographic regions and reduces access and utilization. As a result, many potentially transplantable livers are not matched to recipients in time, underscoring the need for improved preservation strategies.

To address these limitations, substantial effort has been directed toward developing approaches that extend liver preservation time, such as supercooling, partial freezing, and vitrification [9,10,11,12,13,14,15,16,17,18,19,20,21]. In addition to novel preservation methods, liver perfusion protocols, including hypothermic oxygenated machine perfusion (HOPE) and normothermic machine perfusion (NMP), have also been an area of intense research focus [12,15,16,17,18,20,21,22,23,24,25]. Despite these advances that have indeed increased the maximum time livers can be stored, static cold storage (SCS) remains the clinical standard due to its simplicity and accessibility. While effective at slowing cellular metabolism, SCS does not prevent preservation-related injury and can exacerbate cellular damage upon reperfusion, particularly within the liver microvasculature and biliary tract, impairing graft function and post-transplant outcomes [8,22,26,27,28,29,30,31]. Cold ischemia is only tolerated for a limited duration before irreversible damage sets in, largely driven by ischemia-reperfusion injury (IRI) [32], which remains a major challenge in transplantation.

Among the various cell types in the liver, endothelial cells (ECs) play a central role in maintaining vascular integrity, regulating inflammation, and supporting hepatocellular function [8,16,29,33,34,35,36,37,38]. They are also key regulators of reactive oxygen species (ROS) generated during reperfusion following ischemia [39,40,41]. As a result, ECs are particularly susceptible to oxidative injury induced by cold ischemia; damage to these cells disrupts redox homeostasis and contributes to liver dysfunction and graft impairment [42,43]. Liver sinusoidal endothelial cells (LSECs), which are highly specialized and essential for liver functions, are therefore of particular interest in understanding preservation-related vascular injury [8,33,34,44].

Despite extensive studies on liver preservation injury across SCS, machine perfusion, and extended storage approaches [9,10,11,12,13,14,15,16,17,18,22,23,26,35,45,46,47,48,49,50,51], investigations specifically targeting the endothelium, particularly the liver microvasculature, remain limited [17,18,24,25,36,51,52]. One major challenge is that LSECs are difficult to study in whole organ transplant models, where cell-specific responses are often mixed, especially in vivo post-transplantation. As a result, there is a significant gap in understanding how preservation impacts LSECs. This limitation hinders efforts to elucidate the role of LSECs in IRI, including their contributions to reduction capacity, blood flow regulation, coagulation, and inflammatory signaling, all of which are critical for supporting hepatocyte viability following transplantation. Consequently, this knowledge gap restricts the development of targeted improvements in preservation solutions and technologies. Notably, even with advanced preservation methods that extend storage duration, IRI remains a persistent and unresolved challenge [10,11,14,19,20,21]. Hence, targeting endothelial resilience during cold storage may enhance graft viability, extend preservation time, and ultimately increase the number of successful liver transplants.

In this study, we isolated rat LSECs to evaluate the impact of SCS-mediated preservation injury in comparison to hepatocytes and other non-parenchymal cells (NPCs), including hepatic stellate cells (HSCs) and Kupffer cells (KCs). We assessed the effects of SCS on LSEC function, cell death and oxidative stress-related gene expression, and cytokine and chemokine secretion. Finally, we investigated potential therapeutic strategies to mitigate LSEC injury, including the use of small molecules targeting apoptosis and oxidative stress.

## 2. Methods

### 2.1. Hepatocyte Isolation

Primary rat hepatocytes were sourced from the Cell Resource Core at Massachusetts General Hospital (MGH) and were used for all experiments. Hepatocytes were isolated from two- to three-month-old female Lewis rats (Charles River Laboratories, Cambridge, MA, USA), as described previously [9]. Experiments were conducted with cells from isolations resulting in a minimum cell viability of 90%, as determined by trypan blue exclusion or acridine orange and propidium iodide (AOPI) staining with a Cellometer K2 (Nexcelom Bioscience, Lawrence, MA USA). Following isolation, the cells were stored on ice for a maximum of 3 h in seeding medium consisting of Dulbecco’s Modified Eagle Medium (DMEM, Gibco, Grand Island, NY, USA) supplemented with 1% penicillin and streptomycin (P/S, Sigma-Aldrich, Burlington, MA USA), and 10% fetal bovine serum (FBS, Gibco, Grand Island, NY, USA).

### 2.2. Hepatocyte Culture

Plastic 96-well tissue-culture plates were first coated with type I collagen (R&D Systems, Minneapolis, MN, USA) at 15 µg/cm^2^. Then, each well was seeded with hepatocytes at a density of 1.25 × 10^5^ cells/cm^2^ in seeding medium. Following one hour of incubation at standard culture conditions (37 °C, 5% CO_2_) to allow for cell attachment, the medium was changed to a culture medium consisting of Williams’ E Medium (Sigma-Aldrich, Burlington, MA, USA) supplemented with 20 ng/mL epidermal growth factor (EGF) (Sigma-Aldrich, Burlington, MA, USA), 7.5 μg/mL hydrocortisone (Pfizer Inc., New York City, NY, USA), 7 μg/mL glucagon (Boehringer Ingelheim, Ridgefield, CT, USA), 0.5 unit/mL insulin (Eli Lilly, Indianapolis, IN, USA), 1% P/S, and 10% FBS. In the culture medium, the cells are stabilized overnight under standard culture conditions. Subsequent medium changes with supplemented Williams’ E Medium were performed daily.

### 2.3. NPC Isolation

NPCs (HSCs, KCs, and LSECs) were isolated based on differences in density (HSCs) and cell attachment (LSECs and KCs), and isolation was performed using a modified protocol that has previously shown cell separation purity of >90% [53]. Briefly, the NPC fraction from the hepatocyte isolation was spun down at 650× *g* and 4 °C for 10 min and then resuspended in a 17% iodixanol (Sigma-Aldrich, Burlington, MA, USA) solution diluted in Gey’s Balanced Salt Solution (GBSS, Sigma-Aldrich, Burlington, MA, USA). Then, 11.5% iodixanol diluted in GBSS was carefully overlaid on top of the 17% solution, and then GBSS was overlaid on top of the 11.5% solution to create three separate density layers at a 5:5:2 ratio. NPCs were spun down at 1400× *g* and 4 °C for 21 min with no brake on the centrifuge. The top cell fraction with the lowest density (HSCs) was then removed and diluted into the seeding medium described above. The second cell fraction with a higher density (KCs and LSECs) was also removed and diluted in seeding medium. Both cell fractions were then spun down at 650× *g* and 4 °C for 10 min. Following centrifugation, the HSCs were resuspended in seeding medium, and the KC and LSEC fraction was resuspended in Endothelial Growth Medium 2 (EGM-2, Lonza, Walkersville, MD, USA) supplemented with 1% P/S.

### 2.4. NPC Culture

For HSCs and LSECs, plastic 96-well tissue-culture plates were first coated with type I collagen at 15 µg/cm^2^, while KCs were seeded on non-coated plates. After resuspension in seeding medium, HSCs were seeded at a density of 9.0 × 10^5^ cells/cm^2^ and allowed to attach overnight under standard culture conditions. After resuspension in EGM-2, the KC and LSEC fraction was seeded at a density of 2.75 × 10^6^ cells/cm^2^ (combined count of KCs and LSECs) on the non-coated plates and incubated at 37 °C for 5 min to allow the KCs to selectively adhere. Following the incubation, the LSEC fraction was then removed and replaced with seeding medium. The LSEC fraction was seeded on the collagen-coated plates at a density of 1.5 × 10^6^ cells/cm^2^, and both KCs and LSECs were allowed to attach overnight under standard culture conditions. The following morning, all cells were washed, and subsequent medium changes with each cell’s respective medium were performed daily.

### 2.5. SCS and Treatments

Following stabilization in culture (1 d for hepatocytes and LSECs, 3 d for HSCs and KCs), cells underwent SCS. For SCS, the medium was removed and replaced with ice-cold University of Wisconsin solution (UW, Bridge to Life, Duluth, GA, USA). Cells were then stored for up to 3 d at 4 °C prior to recovery. After SCS, cells were rescued by removing the UW solution and performing a wash with ice-cold medium (Williams’ E for hepatocytes, EGM-2 for LSECs, and seeding medium for HSCs and KCs), and then cells were kept in their respective media for 24 or 48 h under standard culture conditions prior to experiments. For fresh samples, hepatocytes and LSECs were cultured for 3 d, and HSCs and KCs were cultured for 5 d prior to conducting assays to be used as controls. The results are reported as 1d and 3d SCS to indicate the duration of SCS.

For SCS LSEC treatments, the UW medium was supplemented with either 5% 35 kDa (Sigma-Aldrich, Burlington, MA, USA) polyethylene glycol (PEG, Sigma-Aldrich, Burlington, MA, USA) to promote membrane stability or an antiapoptotic cocktail consisting of Chroman 1 (0.05 μM, MedChemExpress, Monmouth Junction, NJ, USA), Emricasan (5 μM, Selleck Chemicals, Houston, TX, USA), Polyamines (1:1000 dilution, Sigma-Aldrich, Burlington, MA, USA), and trans integrated stress response inhibitor (0.7 μM, Tocris Bioscience, Minneapolis, MN, USA) (CEPT) to inhibit apoptosis during and immediately following cold ischemia at a previously optimized concentration [15,54]. For recovery LSEC treatments, the EGM-2 following storage was supplemented with an antioxidant concentration of Trolox (5 μM, Cayman Chemicals, Ann Arbor, MI, USA) to prevent oxidative stress following return to normoxia following cold ischemia [55]. Heat shock, to promote production of heat shock proteins, was performed by recovering the cells in 42 °C EGM-2 and incubating at 42 °C for 30 min immediately following SCS, and then storing them at normal culture conditions. For heat shock and PEG exploratory experiments, as well as 3d SCS and 24 h of recovery Trolox caspase 3/7 activity, only two biological replicates were performed. For all treatments, LSECs were cultured for either 24 or 48 h following SCS prior to experiments.

### 2.6. Immunocytochemistry

Cells were fixed in 10% formalin (LabChem, Zelienople, PA, USA) for 30 min at room temperature and then washed 5 times for 5 min each with phosphate-buffered saline (PBS, Genesee Scientific, El Cajon, CA, USA). Cells were then permeabilized by incubating with 0.1% Triton-X (Sigma-Aldrich, Burlington, MA, USA) for 30 min at room temperature and then washed 5 times for 5 min each with PBS. Cells were then blocked in 5% Bovine Serum Albumin (BSA, Sigma-Aldrich, Burlington, MA, USA) diluted in PBS for 2 h at room temperature. After blocking, cells were incubated at 4 °C overnight in albumin (hepatocytes, 1:500 dilution, Abcam, Waltham, MA, USA, RRID: AB_10888110), SE-1 (LSECs, 1:500 dilution, Novus Biologicals, Centennial, CO, USA, RRID: AB_3179491), or CD163 (KCs, 1:200 dilution, Abcam, Waltham, MA, USA, RRID: AB_3751206) primary antibodies diluted in 5% BSA. The next day, all cells were washed 5 times for 5 min each with PBS and then incubated with either goat anti-rabbit Alexa Fluor 594 (for hepatocytes 1:1000 dilution, for KCs 1:500 dilution, Abcam, Waltham, MA, USA, RRID: AB_2650602) or goat anti-mouse Alexa Fluor 594 (for LSECs 1:1000 dilution, Abcam, Waltham, MA, USA, RRID: AB_2650601) secondary antibodies in 5% BSA for 6 h at 4 °C. Following secondary antibody staining, cells underwent 5 min washes with PBS until minimal background was observed. Cells were then placed in mounting medium with DAPI (Ibidi, Gräfelfing-Lochham, Germany). For HSCs, following permeabilization with Triton-X, cells were incubated with Phalloidin-iFluor 594 (1:1000 dilution, Abcam, Waltham, MA, USA) in 1% BSA for 90 min. After the 90 min incubation, cells were washed with PBS and placed in mounting medium with DAPI. All cells were then imaged using a Nikon AXR laser scanning confocal microscope (Nikon, Tokyo, Japan). Post-imaging processing was performed using ImageJ software (Version 1.53, NIH, Bethesda, MD, USA).

### 2.7. Live/Dead Staining

Cell staining was performed using a Live/Dead viability kit (Thermo Fisher, Waltham, MA, USA) consisting of calcein AM and ethidium homodimer stains and prepared as per the manufacturer’s protocols. Images were taken with an EVOS M5000 inverted microscope (Thermo Fisher, Waltham, MA, USA). Post-imaging processing and analysis was performed using ImageJ software. Viability was presented as a percentage of the viability of the fresh group.

### 2.8. Metabolic Activity Assay

To measure metabolic activity, resazurin reduction activity was measured by the PrestoBlue assay (Invitrogen, Waltham, MA, USA). PrestoBlue reagent was added to 37 °C culture medium at a 1:9 ratio to create a working reagent and added to each well. The cells were then incubated at standard culture conditions for 30 min. After 30 min, the working reagent from each well was transferred to a black 96-well plate. The fluorescence (excitation 530 nm; emission 590 nm) was read from the top of the well using a Synergy 2 microplate reader (Biotek, Winooski, VT, USA) per the manufacturer’s protocol to determine cellular resazurin reduction activity. The plain working reagent was measured to determine the background baseline, which was subtracted from the cellular activity reading. Resazurin reduction activity is presented as a percentage of the activity in the fresh group.

### 2.9. ATP Assay

To measure ATP levels, the CellTiter-Glo 2.0 (Promega, Fitchburg, WI, USA) assay reagent was prepared beforehand by a 1:1 combination with culture medium to create a working reagent and was added to each well. The plate was then incubated for 30 min at room temperature while protected from light. Following 30 min, the working reagent from each well was transferred to a white 96-well plate. The luminescence (peak emission 560 nm) was read from the top of the well using the Synergy 2 microplate reader (Biotek, Winooski, VT, USA) per the manufacturer’s protocol. The plain working reagent was measured to determine the background baseline. The ATP levels are presented as a percentage of the ATP levels in the fresh group.

### 2.10. Caspase Activity Assay

To measure caspase 3 and 7 activity, the Caspase-Glo 3/7 (Promega, Fitchburg, WI, USA) assay reagent was prepared beforehand by a 1:1 combination with culture medium to create a working reagent, then added to each well. The plate was incubated for 30 min at room temperature while protected from light. After 30 min, the working reagent from each well was transferred to a white flat-bottom 96-well plate. The luminescence (peak emission 560 nm) was read from the top of the well using a Synergy 2 microplate reader (Biotek, Winooski, VT, USA) per the manufacturer’s protocol. The plain working reagent was measured to determine the background baseline. To better measure the caspase 3/7 activity levels of adherent cells, caspase 3/7 activity was normalized to cellular viability at each timepoint and is presented as the fold change compared to the fresh group.

### 2.11. ROS Production Assay

LSECs were seeded in clear-bottom black 96-well plates and cultured as described above. To determine ROS production, H2DCFDA (Tocris, Minneapolis, MN, USA) was diluted in EGM-2 to a concentration of 50 μM to create a working reagent. Cell culture medium was then replaced with the working reagent and incubated at standard conditions for 30 min while protected from light. Following the incubation, the fluorescence (excitation of 492 nm and emission of 522 nm) was read from the bottom of the well using a Spectramax iD3 (Molecular Devices, San Jose, CA, USA). For positive controls, H_2_O_2_ (1 mM, Sigma-Aldrich, Burlington, MA, USA) was added to the working reagent prior to incubation. The plain working reagent and the H_2_O_2_-supplemented working reagent were also measured to determine the experimental and positive controls’ respective background baselines. To better measure the ROS production of adherent LSECs, ROS production was normalized to cellular viability at each timepoint and is presented as the fold change compared to the fresh group.

### 2.12. Acetylated Low-Density Lipoprotein (LDL) Uptake

DiI-conjugated acetylated LDL (DiI-Ac-LDL) staining solution (Cell Applications, San Diego, CA, USA) was added to each well of LSECs at a 1:10 ratio and gently mixed into the culture medium as per the manufacturer’s protocol. The LSECs were then incubated for 4 h under standard culture conditions. Following incubation, the Dil-Ac-LDL-supplemented medium was removed and replaced with Hoechst nuclear stain (1:2000, Thermo Fisher Scientific, Waltham, MA, USA) diluted in PBS and incubated for 5 min at room temperature. Afterwards, the cells were washed once with PBS for 5 min at room temperature and then imaged using an EVOS M5000 inverted microscope. Ac-LDL uptake was determined as red fluorescence intensity normalized to the number of cells in each image’s field of view. Post-imaging processing and analysis was performed using NIH ImageJ software.

### 2.13. Permeability Assay

LSECs were isolated as described above and seeded on 0.4 μm-pore transwell inserts (Corning, Corning, NY, USA). After seeding, cells underwent SCS preservation and treatments as described above. To measure permeability, 4 kDa fluorescein isothiocyanate dextran (FITC-dextran, MilliporeSigma, Burlington, MA, USA) was diluted into EGM-2 to a concentration of 1 mg/mL and transferred to the transwell insert. Fresh EGM-2 medium without FITC-dextran was added to the well below the transwell insert, and LSECs were incubated for 30 min under standard culture conditions. Following incubation, 100 μL of medium below the transwell insert was transferred to a black 96-well plate, and the fluorescence was measured (excitation of 485 nm and emission of 525 nm) from the top of the well using a Spectramax iD3 (Molecular Devices, San Jose, CA, USA). An unseeded transwell was used as a 100% permeability control, and EGM-2 was used as a 0% permeability control to measure permeability.

### 2.14. Heparin Attachment Assay

Fluorescein-conjugated heparin (Thermo Fisher Scientific, Waltham, MA, USA) was diluted in EGM-2 (1:100) and was added to the LSECs or empty wells and incubated under standard culture conditions for 24 h. Afterwards, the medium was transferred to a black 96-well plate, and the fluorescence of the unattached heparin was measured (excitation of 485 nm and emission of 525 nm) using a Spectramax iD3 (Molecular Devices, San Jose, CA, USA). EGM-2 without fluorescein-conjugated heparin was used as a 100% attachment control, and fluorescein-conjugated heparin added to empty wells was used as a 0% attachment control. To better measure the heparin attachment of adherent LSECs, heparin attachment is normalized to LSEC viability at each respective measurement point.

### 2.15. Cytokine and Chemokine Multiplex Immunoassay Array

LSEC medium was collected, snap-frozen and shipped to Eve Technologies (Calgary, AB, Canada). The cytokine and chemokine levels were then measured using the Rat Cytokine/Chemokine 27-Plex Discovery Assay Array (Eve Technologies, Calgary, AB, Canada); samples were run in two technical replicates. All samples that were below the observable range were treated as having a cytokine concentration of 0 pg/mL. To better measure the cytokine and chemokine production of adherent LSECs, cytokine and chemokine expression is normalized to LSEC viability at each respective measurement point.

### 2.16. Transmission Electron Microscopy (TEM) Sample Preparation and Imaging

LSECs were isolated and seeded on transwell inserts as described above. Afterwards, cells underwent SCS and treatments as described above. After the experiments, LSECs were fixed (within the transwell plates in which they were cultured) using 2.0% paraformaldehyde/2.5% glutaraldehyde in 0.1 M sodium cacodylate buffer (pH 7.4) for 3–4 h at room temperature on a gentle rotator; the fixative was replaced with fresh fixative solution and allowed to further infiltrate overnight at 4 °C.

Membranes were rinsed several times in 0.1 M sodium cacodylate buffer, then excised as a whole from their transwell supports, placed into 1% osmium tetroxide for 1 h at room temperature on a gentle rotator, then again rinsed several times in cacodylate buffer. Each membrane was then cut into 3 smaller pieces (for easier embedding) and dehydrated through a graded series of ethanol to 100%. Specimens were then transferred into a 2:1 mix of 100% Ethanol:Eponate resin (Ted Pella, Redding, CA, USA), allowed to infiltrate for 2 h at room temperature on a gentle rotator, and then placed into a 1:1 mix of 100% Ethanol:Eponate resin for overnight infiltration, again at room temperature on a gentle rotator.

The following day, specimens were transferred into fresh 100% Eponate and allowed to infiltrate for several hours on a gentle rocker table, then embedded in flat molds with fresh 100% Eponate resin and allowed to polymerize in a 60 °C oven (24–48 h).

Ultra-thin (70 nm) sections were cut from embedded blocks using an Ultra 45° diamond knife (DIATOME/EMS, Hatfield, PA, USA) and a Leica EM UC7 ultramicrotome, collected onto formvar-coated grids, stained with 2% uranyl acetate and Reynold’s lead citrate and examined in a JEOL JEM 1011 transmission electron microscope at 80 kV. Images were collected using an AMT digital imaging system with proprietary image capture software (Advanced Microscopy Techniques, Woburn, MA, USA).

### 2.17. Cell Death and Oxidative Stress Gene Expression Assay

Total RNA was isolated from LSECs using the RNeasy Mini Kit (Qiagen, Germantown, MD, USA). Following initial isolation, RNA clean-up and concentration was performed using the Norgen Biotek RNA Clean-Up and Concentration Kit (Norgen Biotek, Thorold, ON, Canada). Afterwards, complementary DNA (cDNA) was synthesized using the RT^2^ First Strand Kit (Qiagen, Germantown, MD, USA), and the gene expression of the LSECs was measured using the RT^2^ Profiler PCR Array Rat Cell Death PathwayFinder (Qiagen, Germantown, MD, USA) and the RT^2^ Profiler PCR Array Rat Oxidative Stress (Qiagen, Germantown, MD, USA) kits per the manufacturer’s instructions. Gene expression was calculated using the delta delta CT method, with the RT^2^ Profiler PCR Array housekeeping genes being utilized. Gene expression of treated LSECs was calculated as a fold change to fresh LSECs. For PCR arrays, a technical replicate of one was performed.

### 2.18. Statistical Analysis

**Figure 1:**
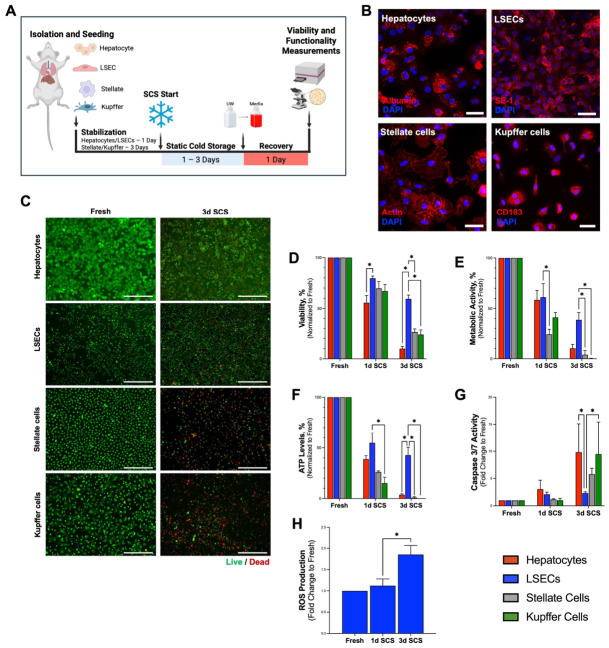
Isolated rat LSECs have a different response to SCS than other hepatic cells. (**A**) Schematic of rat hepatic cell isolation, culture and SCS. (**B**) Immunostaining images of hepatocytes (**top left**), LSECs (**top right**), stellate (**bottom left**), and Kupffer (**bottom right**) cells following isolation. (**C**) Representative live/dead images of fresh (**left**) and 3d SCS (**right**) hepatic cells. (**D**) Viability. (**E**) Metabolic activity. (**F**) ATP levels. (**G**) Caspase activity of fresh, 1d SCS, and 3d SCS hepatic cells. (**H**) ROS production of fresh, 1d SCS, and 3d SCS LSECs. Scale bars (**B**) 40 μm and (**C**) 300 μm. * denotes *p* < 0.05. Sample size: (**D**) hepatocytes *n* = 3 for all, LSEC fresh *n* = 8, LSEC 1d SCS *n* = 5, LSEC 3d SCS *n* = 9, stellate cells *n* = 5 for all, Kupffer cells *n* = 6 for all; (**E**) hepatocytes *n* = 3 for all, LSEC fresh *n* = 18, LSEC 1d SCS *n* = 7, LSEC 3d SCS *n* = 16, stellate cells *n* = 3 for all, Kupffer cells *n* = 3 for all; (**F**) hepatocytes *n* = 3 for all, LSEC fresh *n* = 17, LSEC 1d SCS *n* = 6, LSEC 3d SCS *n* = 15, stellate cells *n* = 3 for all, Kupffer cells *n* = 3 for all; (**G**) hepatocytes *n* = 3 for all, LSEC fresh *n* = 15, LSEC 1d SCS *n* = 7, LSEC 3d SCS *n* = 15, stellate cells *n* = 3 for all, Kupffer cells *n* = 3 for all; (**H**) *n* = 3 for all.

**Figure 2:**
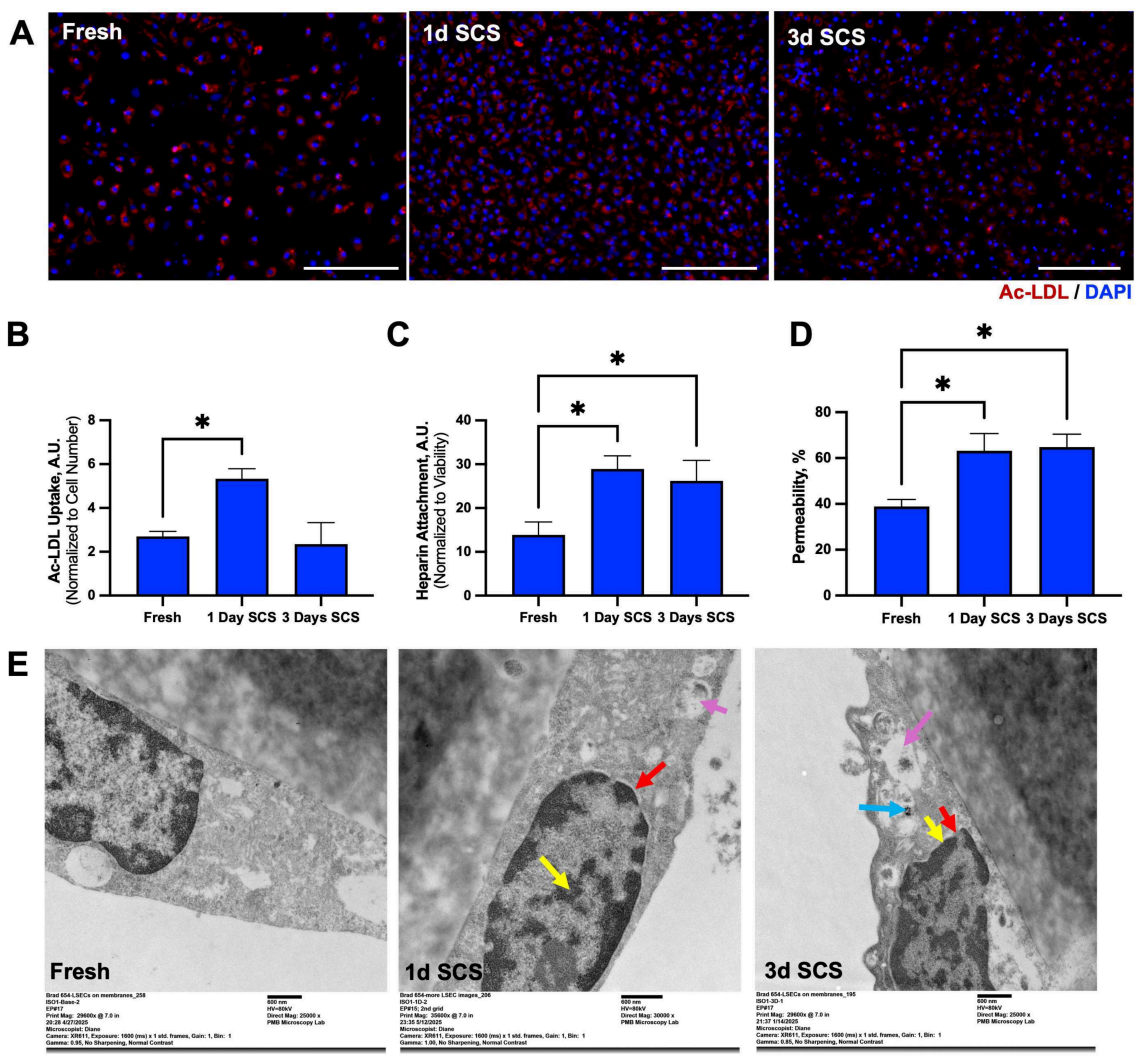
LSECs lose functionality following SCS. (**A**) Representative images of fresh, 1d SCS, and 3d SCS LSECs stained for Ac-LDL. (**B**) Ac-LDL uptake. (**C**) Heparin attachment and (**D**) permeability of fresh, 1d SCS, and 3d SCS LSECs. (**E**) Representative TEM image of fresh (**left**), 1d SCS (**middle**), and 3d SCS (**right**) LSECs with nuclear membrane separation (red arrows), chromatin aggregation (yellow arrows), phagosomal content (blue arrows), and lipid content (purple arrows) indicated. Scale bars (**A**) 300 μm and (**E**) 600 nm. * denotes *p* < 0.05. Sample size: (**B**) *n* = 3 for all, (**C**) *n* = 8 for all, (**D**) *n* = 6 for all.

**Figure 3:**
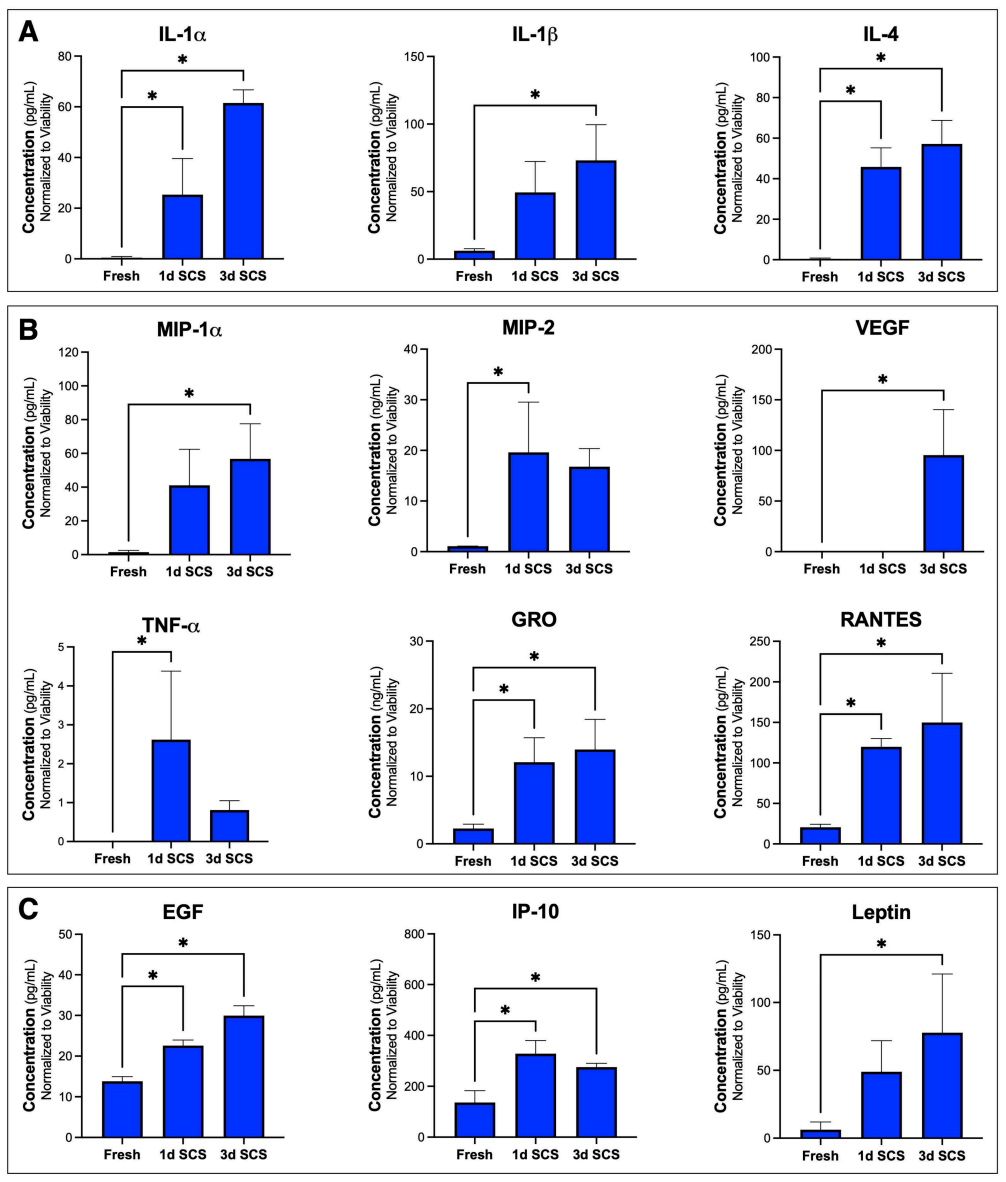
SCS leads to an increase in LSEC chemokine and cytokine secretion. Levels of (**A**) interleukin, (**B**) inflammatory, and (**C**) general chemokines and cytokines secreted by fresh, 1d SCS, and 3d SCS LSECs. * denotes *p* < 0.05. Sample size: *n* = 3 for all.

**Figure 4:**
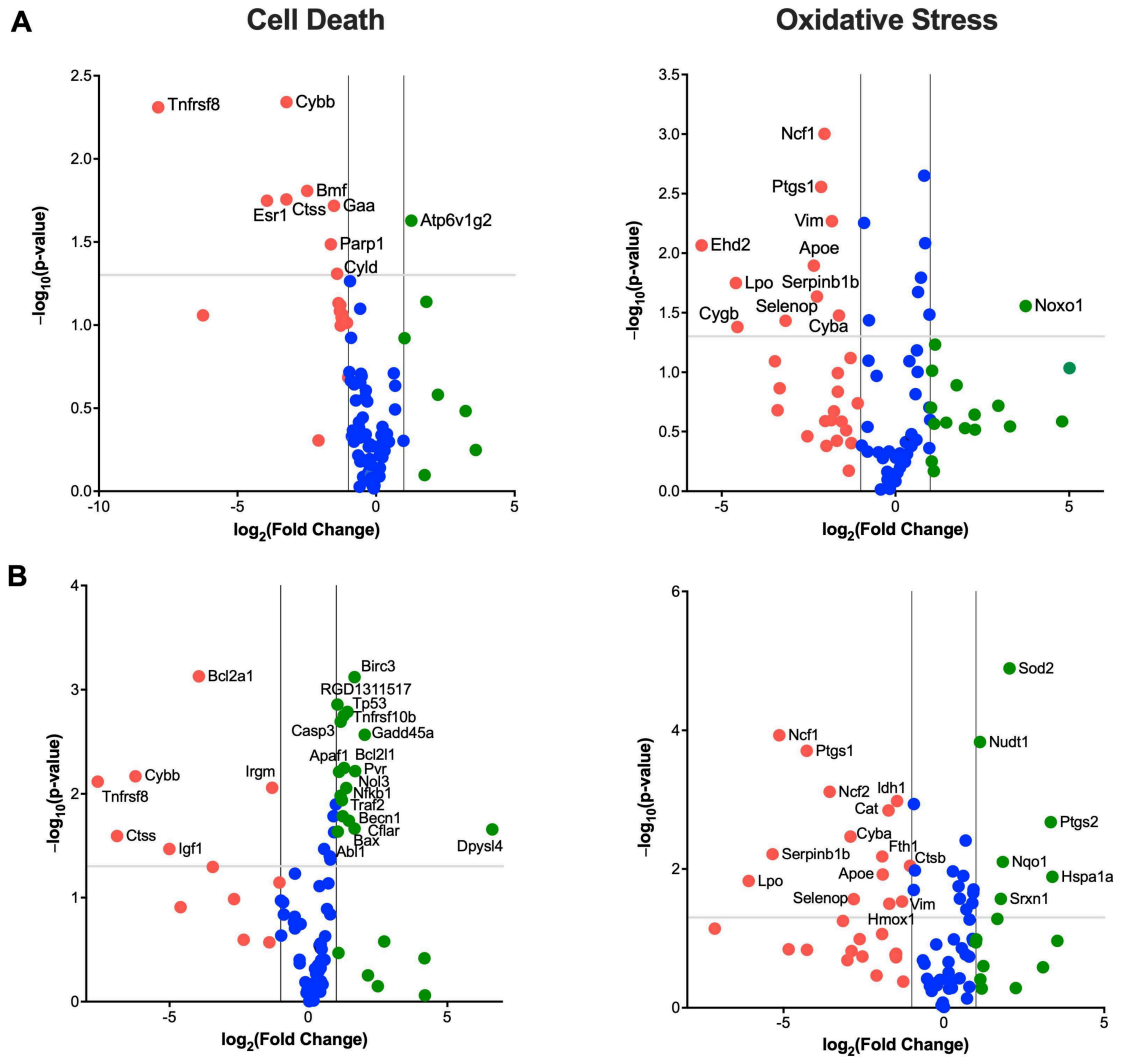
SCS leads to an increase in both cell death and oxidative stress-related gene expression. Changes (down regulation = red, upregulation = green, and no change = blue) in cell death (**left**) and oxidative stress (**right**) gene expression of LSECs following (**A**) 1d SCS or (**B**) 3d SCS when compared to fresh samples. Sample size: Cell Death Fresh *n* = 3, Cell Death 1d SCS *n* = 4, Cell Death 3d SCS *n* = 3, Oxidative Stress Fresh *n* = 4, Oxidative Stress 1d SCS *n* = 3, Oxidative Stress 3d SCS *n* = 3.

**Figure 5:**
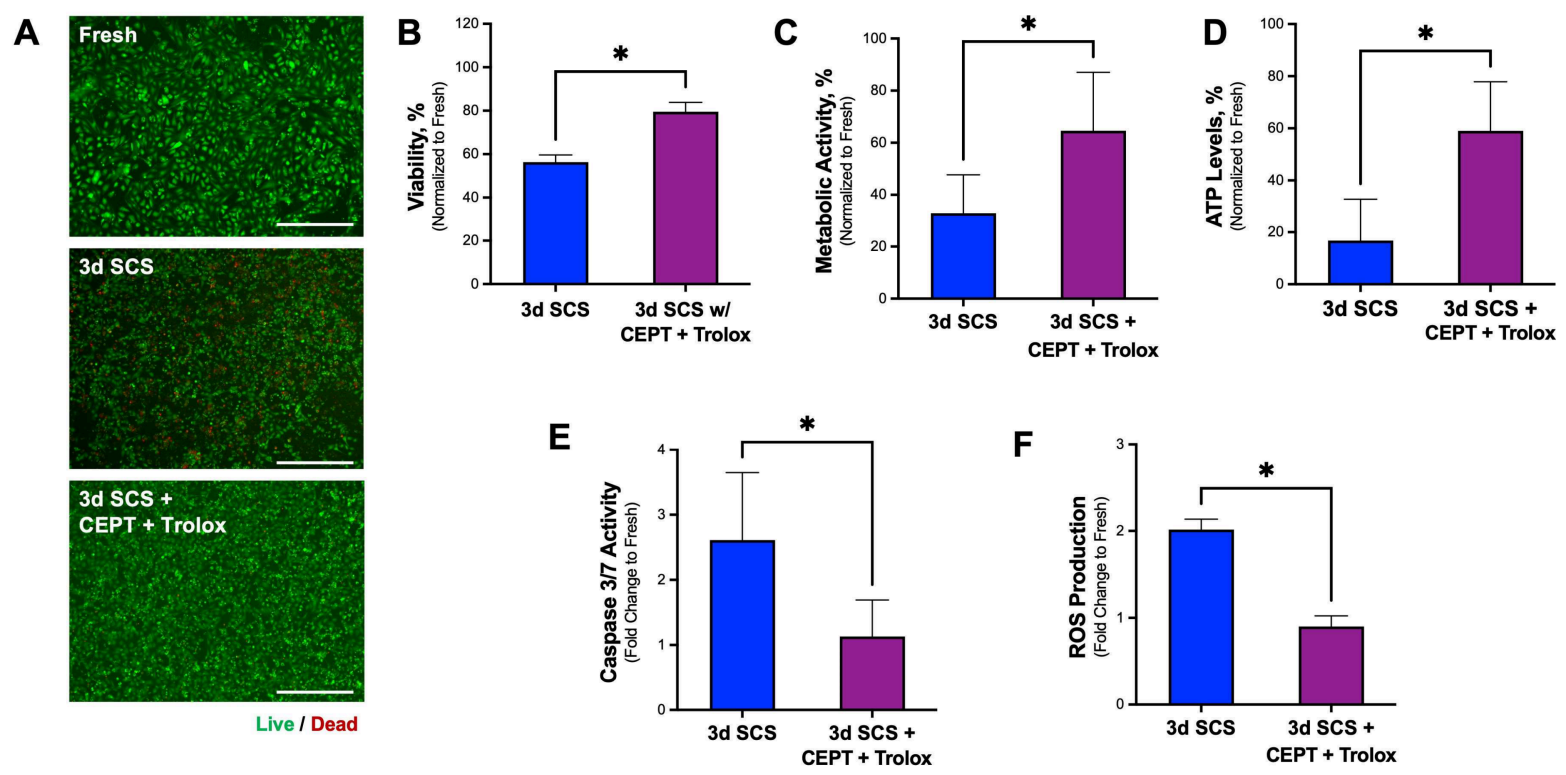
Combined treatment of CEPT and Trolox can rescue LSECs from preservation injury. (**A**) Representative live/dead images of fresh (**top**), 3d SCS (**middle**), and 3d SCS + CEPT + Trolox LSECs (**bottom**). (**B**) Viability. (**C**) Metabolic activity. (**D**) ATP levels. (**E**) Caspase activity and (**F**) ROS production of 3d SCS LSEC either with or without CEPT + Trolox. Scale bar = 300 μm. * denotes *p* < 0.05. Sample size: (**B**) *n* = 3 for all, (**C**) 3d SCS *n* = 4, 3d SCS w/CEPT + Trolox *n* = 5, (**D**) 3d SCS *n* = 3, 3d SCS w/CEPT + Trolox *n* = 4, (**E**) *n* = 5 for all, (**F**) *n* = 3 for all.

**Figure 6:**
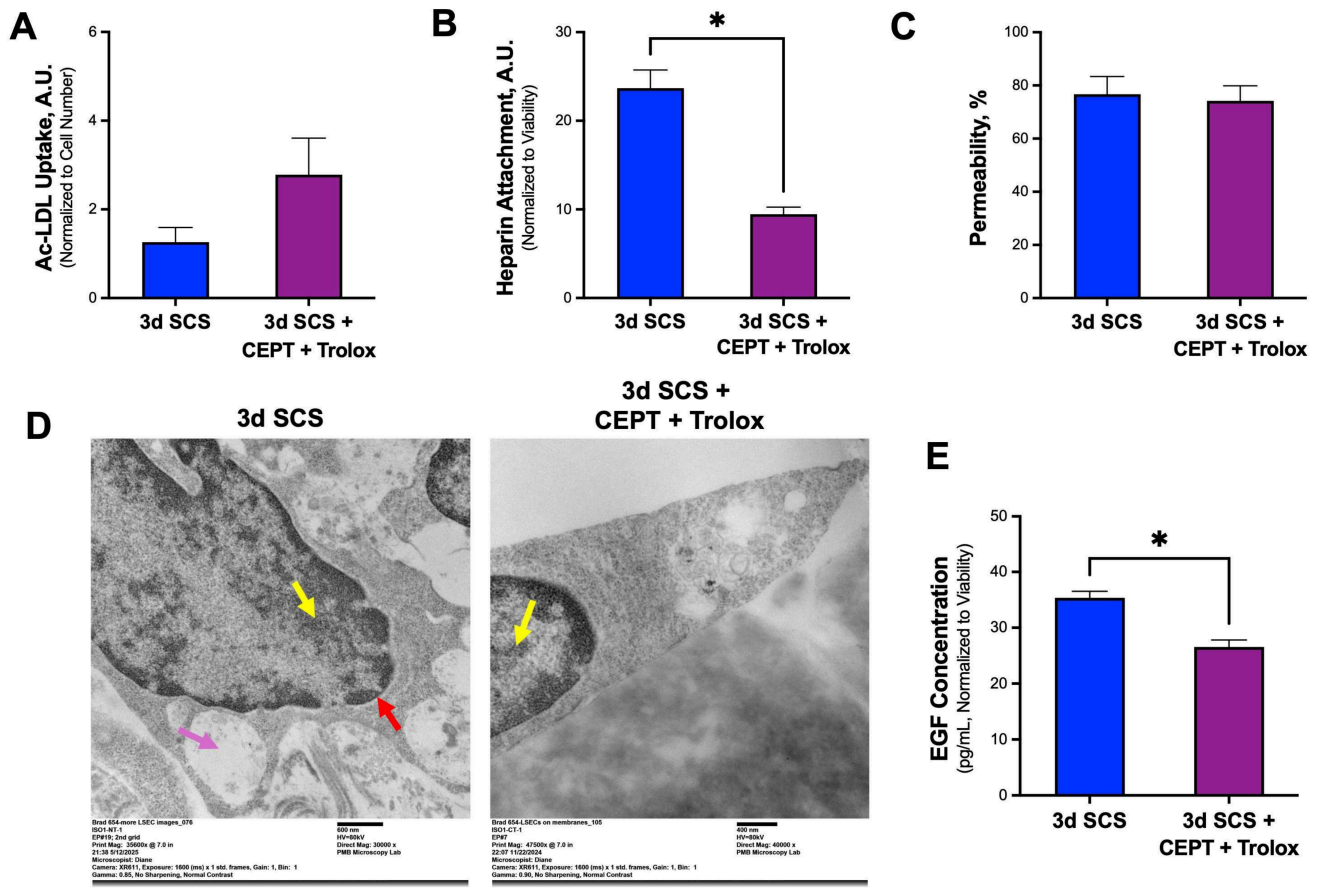
CEPT and Trolox treatment has mixed beneficial effect on LSEC functionality following 3 days SCS and 48 h of recovery. (**A**) Ac-LDL uptake, (**B**) heparin attachment, and (**C**) permeability of 3d SCS and 3d SCS + CEPT + Trolox LSECs. (**D**) Representative TEM images of 3d SCS and 3d SCS + CEPT + Trolox LSECs with nuclear membrane separation (red arrows), chromatin aggregation (yellow arrows), and lipid content (purple arrows) indicated (**E**) EGF secretion levels of 3d SCS and 3d SCS + CEPT + Trolox LSECs. Scale bars (**D**, **left**) 600 nm (**D**, **right**) 400 nm. * denotes *p* < 0.05. Sample size: (**A**) *n* = 3 for all, (**B**) *n* = 3 for all, (**C**) *n* = 4 for all, (**E**) *n* = 3 for all.

The data for the figures is presented as mean ± the standard deviation (SD). For Figure 1, Figure 2 and Figure 3 and Supplemental Figures S1–S3, one-way analysis of variance (ANOVA) with either a Tukey’s (Figure 1) or Dunnett’s (Figure 2 and Figure 3 and Supplemental Figures S1–S3) post hoc test was performed. For Figure 4, Figure 5 and Figure 6, Table 1 and Table 2, Supplemental Tables S1 and S2, and Supplemental Figures S4 and S5, an unpaired two-tailed Student’s *t*-test was performed. All *p* values are two-sided, and statistical significance was defined as *p* lower than 0.05. Each individual isolation was treated as a biological replicate, and sample size (indicated as ‘*n*’ throughout the rest of the manuscript) was greater than or equal to 3 for all experiments unless otherwise stated, with individual sample sizes indicated in the figure legends. Technical replicates (i.e., wells) from each isolation were also greater than or equal to 3 for all experiments unless otherwise stated. All statistical analyses were performed in Prism 10 (GraphPad Software Inc., San Diego, CA, USA). 

## 3. Results

### 3.1. Extended SCS and Subsequent Recovery Is Detrimental to NPCs

In order to determine cell-specific responses to SCS, we isolated rat hepatocytes, LSECs, HSCs, and KCs from adult rats and subjected all cell types to up to 3 days of SCS (Figure 1A) to cause significant preservation injury. All cell types retained their canonical markers in culture (Figure 1A,B). We found that with 1d SCS, all cell types maintained a viability greater than 50%, with hepatocytes having a significantly lower viability than LSECs (Figure 1C,D). When the length of SCS was extended to 3d, however, LSECs were the only cell type of all isolated cells to maintain a viability greater than 50% (Figure 1C,D). When looking at an additional viability indicator, metabolic activity via resazurin reduction, we saw that, similarly, at 1d SCS, LSECs maintained significantly higher metabolic activity than HSCs, with LSECs having higher metabolic activity than both HSCs and KCs following 3d SCS (Figure 1E). We also saw additional evidence of these viability differences with ATP levels and observed that LSECs 1d SCS had higher ATP levels than KCs; however, when storage was extended to 3d SCS, LSECs maintained significantly higher ATP levels than all other cell types (Figure 1F). The only significant differences measured in caspase 3/7 activity were following 3d SCS, with hepatocytes and KCs exhibiting significantly higher caspase 3/7 activity levels than LSECs, while all cell types showed an increase in caspase 3/7 activity levels when compared to their fresh counterparts (Figure 1G). Finally, we measured an increase in ROS production in LSECs following 3d SCS (Figure 1H). As LSECs were seen to have greater viability, metabolic activity, and ATP levels in addition to having lower caspase 3/7 activity, indicating a greater potential for rescue from preservation injury, we further characterized the effect of SCS on LSECs and explored potential treatment strategies for their recovery.

### 3.2. Extended SCS and Subsequent Recovery Has a Detrimental Effect on LSEC Function

In addition to measuring LSEC viability and metabolic activity, we also investigated the effect of 1d SCS and 3d SCS on endothelial-specific functions. We found that 1d SCS led to a significant increase in scavenging functions measured by Ac-LDL uptake and heparin attachment (Figure 2A–C). However, with 3d SCS, we saw that this increase was only maintained for heparin (Figure 2C), while Ac-LDL uptake decreased to the levels observed in fresh samples. We also utilized transwell membranes to measure the changes in LSEC permeability brought about by SCS (Figure 2D), with both 1d and 3d SCS significantly increasing LSEC permeability when compared to freshly isolated LSECs. To further explore whether SCS had any effect on LSEC morphology, we used transmission electron microscopy (TEM) to observe any changes in LSEC ultrastructure. We observed that SCS led to dramatic changes in LSEC phenotype, including an increase in cell damage indicators such as nuclear membrane separation, chromatin aggregation, and increases in phagosomal and lipid droplet content (Figure 2E), which appeared to increase as SCS length increased.

As we previously observed an increase in ROS production in LSECs following SCS (Figure 1H), we decided to investigate whether SCS led to any changes in cytokine or chemokine production (Figure 3 and Figure S1). We measured an increase in interleukins IL-1α and IL-4 in 1d SCS, with this increase also being observed in 3d SCS with the addition of IL-1β (Figure 3A). We also observed significant increases in the production of inflammatory cytokines MIP-1α (3d SCS only), MIP-2 (1d SCS only), VEGF (3d SCS only), TNF-α (1d SCS only), GRO, and RANTES (Figure 3B). Additionally, we observed that cell growth stimulation protein EGF, chemoattractant IP-10, and hormone leptin (3d SCS only) underwent increases in production in both 1d SCS and 3d SCS groups (Figure 3C). We observed no significant changes in IFN-γ, IL-5, IL-6, IL-10, IL-13, IL-18, LIX, or MCP-1 secretion (Figure S1).

### 3.3. Recovery from Extended SCS Causes Increases in LSEC Cell Death and Oxidative Stress Gene Expression

To further elucidate which cell death pathways (e.g., apoptosis, autophagy, necrosis) were prevalent in LSEC preservation injury, we used PCR arrays to measure the gene expression changes immediately following SCS and after 24 h of recovery. Interestingly, 1d SCS (with and without recovery), along with 3d SCS without recovery, only led to minor transcriptional changes (Figure 4, Table 1 and Table S1). In contrast, 3d SCS followed by 24 h of recovery led to substantial upregulation of pro-apoptotic genes (Figure 4 and Table 1). Most notable changes were significant upregulation of Abl1, Apaf1, Cflar, Gadd45a, Nol3, Tnfrsf10b, Bax, Casp3, and Tp53. Changes in necrosis- and autophagy-related genes were also observed (Table 1) but involved fewer genes, suggesting that apoptosis rather than autophagy or necrosis is the predominant mode of cell death in LSECs following preservation injury.

**Table 1:** Changes in cell death-related gene expression in 1d SCS and 3d SCS groups 24 h post-recovery.

Genes			Regulation Change *24 h Post-Recovery*	
Symbol	Name	Role	1d SCS	3d SCS
*Bcl2a1*	BCL2 Related Protein A1	*Anti-Apoptosis*	nc	↓*
*Birc3*	Baculoviral IAP Repeat Containing 3	*Anti-Apoptosis*	nc	↑*
*Traf2*	TNF Receptor Associated Factor 2	*Anti-Apoptosis*	nc	↑*
*Bcl2l1*	BCL2 Like 1	*Anti-Apoptosis, Autophagy*	nc	↑*
*Becn1*	Beclin 1	*Autophagy*	nc	↑*
*Ctss*	Cathepsin S	*Autophagy*	↓*	↓*
*Esr1*	Estrogen Receptor 1	*Autophagy*	↓*	nc
*Gaa*	Alpha Glucosidase	*Autophagy*	↓*	nc
*Igf1*	Insulin Like Growth Factor 1	*Autophagy*	nc	↓*
*Irgm*	Immunity Related GTPase M	*Autophagy*	nc	↓*
*Nfkb1*	Nuclear Factor Kappa B Subunit 1	*Autophagy*	nc	↑*
*Bmf*	Bcl2 Modifying Factor	*Necrosis*	↓*	nc
*Cybb*	Cytochrome B-245 Beta Chain	*Necrosis*	↓*	↓*
*Dpysl4*	Dihydropyrimidinase Like 4	*Necrosis*	nc	↑*
*Parp1*	Poly(ADP-Ribose) Polymerase 1	*Necrosis*	↓*	nc
*Pvr*	PVR Cell Adhesion Molecule	*Necrosis*	nc	↑*
*RGD1311517*	N-acetylglucosamine-1-phosphate transferase	*Necrosis*	nc	↑*
*Tnfrsf8*	TNF Receptor Superfamily Member 8	*Necrosis*	↓*	↓*
*Abl1*	ABL Proto-Oncogene 1	*Pro-Apoptosis*	nc	↑*
*Apaf1*	Apoptotic Peptidase Activating Factor 1	*Pro-Apoptosis*	nc	↑*
*Cflar*	CASP8 And FAFF Like Apoptosis Regulator	*Pro-Apoptosis*	nc	↑*
*Gadd45a*	Growth Arrest and DNA Damage Inducible Alpha	*Pro-Apoptosis*	nc	↑*
*Nol3*	Nucleolar Protein 3	*Pro-Apoptosis*	nc	↑*
*Tnfrsf10b*	TNF Receptor Superfamily Member 10b	*Pro-Apoptosis*	nc	↑*
*Bax*	BCL2 Associated X	*Pro-Apoptosis, Autophagy*	nc	↑*
*Casp3*	Caspase 3	*Pro-Apoptosis, Autophagy*	nc	↑*
*Tp53*	Tumor Protein P53	*Pro-Apoptosis, Autophagy*	nc	↑*
*Atp6v1g2*	ATPase H+ Transporting V1 Subunit G2	*Pro-Apoptosis, Necrosis*	↑*	nc
*Cyld*	CYLD lysine 63 Deubiquitinase	*Pro-Apoptosis, Necrosis*	↓*	nc

nc: no change, **↓**: downregulated, ↑: upregulated as compared to fresh * *p* < 0.05.

We also measured the changes in oxidative stress gene expression and observed again that immediately following 1d SCS, there were very few changes in gene expression (Table S2). However, following recovery, we did observe a decrease in the expression of several genes, with most of the changes being the downregulation of ROS metabolism, antioxidants, and oxygen transport-related genes (Figure 4 and Table 2). With increasing SCS preservation to 3 d, we found downregulation of a few genes immediately following storage, with most of them being ROS metabolism-related (Table S2). However, following 24 h of recovery, we saw the upregulation of ROS metabolism-related genes Sod2, Nudt1, Nqo1, and Hspa1a, as well as antioxidant-related genes Ptgs2 and Srxn1 (Figure 4 and Table 2). Interestingly, we also observed the downregulation of many other ROS metabolism, antioxidant, and oxygen transport-related genes, suggesting significant oxidative stress (Figure 4 and Table 2).

**Table 2:** Changes in oxidative stress-related gene expression in 1d SCS and 3d SCS groups 24 h post-recovery.

Gene			Regulation Change *24 h Post-Recovery*	
Symbol	Name	Role	1d SCS	3d SCS
*Ehd2*	EH Domain Containing 2	*Antioxidant (TPx)*	↓*	nc
*Srxn1*	Sulfiredoxin 1	*Other Antioxidants*	nc	↑*
*Lpo*	Lactoperoxidase	*Other Peroxidases*	↓*	↓*
*Ptgs1*	Prostaglandin-Endoperoxide Synthase 1	*Other Peroxidases*	↓*	↓*
*Ptgs2*	Prostaglandin-Endoperoxide Synthase 2	*Other Peroxidases*	nc	↑*
*Serpinb1b*	Serpin Family B Member 1	*Other Peroxidases*	↓*	↓*
*Apoe*	Apolipoprotein E	*Oxidative Stress Responsive*	↓*	↓*
*Fth1*	Ferritin Heavy Chain 1	*Oxidative Stress Responsive*	nc	↓*
*Hmox1*	Heme Oxygenase 1	*Oxidative Stress Responsive*	nc	↓*
*Hspa1a*	Heat Shock Protein Family A (Hsp70) Member 1A	*Oxidative Stress Responsive*	nc	↑*
*Idh1*	Isocitrate Dehydrogenase (NADP(+))1	*Oxidative Stress Responsive*	nc	↓*
*Nqo1*	NAD(P)H Quinone Dehydrogenase 1	*Oxidative Stress Responsive*	nc	↑*
*Nudt1*	Nudix Hydrolase 1	*Oxidative Stress Responsive*	nc	↑*
*Selenop*	Selenoprotein P	*Oxidative Stress Responsive*	↓*	↓*
*Cat*	Catalase	*Oxidative Stress Responsive, Other Peroxidases*	nc	↓*
*Ctsb*	Cathepsin B	*Oxidative Stress Responsive, Other Peroxidases*	nc	↓*
*Cygb*	Cytochrome B-245 Beta Chain	*Oxygen Transporters*	↓*	nc
*Vim*	Vimentin	*Oxygen Transporters*	↓*	↓*
*Sod2*	Superoxide Dismutase 2	*ROS SOD*	nc	↑*
*Cyba*	Cytochrome B-245 Alpha Chain	*ROS SOD (Other Genes)*	↓*	↓*
*Ncf1*	Neutrophil Cytosolic Factor 1	*ROS SOD (Other Genes)*	↓*	↓*
*Ncf2*	Neutrophil Cytosolic Factor 2	*ROS SOD (Other Genes)*	nc	↓*
*Noxo1*	NADPH Oxidase Organizer 1	*ROS SOD (Other Genes)*	↑*	nc

nc: no change, **↓**: downregulated, ↑: upregulated as compared to fresh * *p* < 0.05.

### 3.4. The Addition of CEPT and Trolox Allows LSECs to Survive Extended Preservation Times

To mitigate the preservation-related damage observed in LSECs, we tried multiple treatments both during SCS and recovery, including PEG to promote membrane stability, heat shock to induce production of protective heat shock proteins, anti-apoptotic cocktail CEPT to inhibit apoptosis, and cell-permeable antioxidant Trolox to inhibit oxidative stress. We chose 3d SCS, as this is where we observed the greatest damage to LSECs in vitro, as well as changes in cytokine and gene expression. We observed that the addition of PEG during storage and/or applying heat shock immediately following storage had no effect on LSEC metabolic activity, ATP levels, or caspase 3/7 activity (Figure S2). However, when LSECs were treated with CEPT during SCS and Trolox during 24 h of recovery, we observed a beneficial effect from each treatment individually, as well as both treatments combined (Figure S3). When the combined treatment was extended to 48 h of recovery, there were significant increases in viability, metabolic activity, and ATP levels (Figure 5A–D). We also found that CEPT individually and combined with Trolox significantly lowered caspase 3/7 activity levels (Figure 5E and Figure S3E), and CEPT and Trolox individually and combined lowered ROS production (Figure 5F and Figure S3F) after 24 h and 48 h of recovery.

### 3.5. CEPT and Trolox Have a Beneficial Effect on LSEC Functions and Cytokine/Chemokine Expression

We further investigated the effect of the combined CEPT and Trolox treatment on endothelial-specific functions and chemokine/cytokine production. The addition of CEPT during storage and Trolox during the 48 h of recovery led to a non-significant increase in Ac-LDL uptake (Figure 6A) and a significant decrease in heparin attachment (Figure 6B), suggesting some beneficial effect. However, no changes were observed in LSEC permeability with treatment (Figure 6C). Additionally, TEM revealed that treatment with CEPT and Trolox drastically mitigated the damage indicators previously observed in 1d SCS and 3d SCS (Figure 2E and Figure 6D). We observed very few changes in either chemokine or cytokine production (Figures S4 and S5), with the only significant change being a decrease in cell growth stimulation protein EGF (Figure 6E). These results suggest that treatment with CEPT and Trolox increases LSEC survivability following extended SCS, but its effect on LSEC functionality needs further improvement.

## 4. Discussion

LSECs are known to play a critical role in liver disease, particularly in IRI due to their function as a protective interface between circulation and hepatic tissue [18,26,35,37,38]. Despite this, their role in preservation-induced injury remains poorly understood [17,24,25,26,35,36,38,51,53]. LSECs are highly susceptible to injury during IRI and SCS, undergoing capillarization that disrupts the liver sinusoidal architecture and promotes a profibrotic, vasoconstrictive phenotype [17,18,26,37,51,56]. These changes can contribute to widespread graft dysfunction and failure. However, most prior studies have largely been conducted at the whole-organ level, limiting the ability to resolve LSEC-specific responses to preservation injury. In this study, we address this gap using an LSEC-specific in vitro preservation model to evaluate the effects of SCS on LSECs, identify mechanisms of injury, and assess potential reversibility. Though the length of SCS in our model, particularly 3d SCS, is significantly longer than clinical practice, the goal of our in vitro model was to model preservation damage and loss of functionality, not clinical timing. Further, rat livers have frequently been shown to have longer viable preservation times than human livers [11,14,57,58,59]. As such, we chose 1d SCS and 3d SCS as our preservation lengths, as they provided sufficient damage in our model with significant loss of viability and functionality that allowed the effect of our therapeutic interventions to be clearly observed. Our findings demonstrate that inhibition of apoptosis during SCS using CEPT [15], combined with reduction of oxidative stress during recovery using Trolox, significantly improves LSEC viability after extended SCS. These results highlight the central role of oxidative stress and apoptosis in LSEC injury and suggest that endothelial-targeted interventions may represent a promising avenue to mitigate preservation injury and improve graft utilization.

We first compared the behavior of LSECs under SCS conditions with isolated hepatocytes, HSCs, and KCs. After 1d SCS, all cell types showed some recovery, with viability greater than 50%. However, when SCS length was extended to 3 d, LSECs maintained viability similar to that at 1 d, while hepatocytes, HSCs and KCs exhibited significantly reduced viability, metabolic activity, and ATP levels. Additionally, all cell types showed a trend of increased levels of apoptosis markers caspase 3 and 7. We attribute this to the fact that endothelial cells possess defense mechanisms against oxidative stress [60], a key component of preservation injury in SCS [32,61]. Given these defense mechanisms, which provide LSECs with a greater potential for rescue, along with their previously mentioned critical role in IRI protection for other hepatic cells and lack of cell-specific studies, we decided to focus our study more closely on the mechanism of preservation injury on LSECs specifically.

Besides survival, we also examined functional changes in LSECs with varying SCS durations. We observed an increase in ROS production as expected, as well as notable changes in Ac-LDL uptake and heparin scavenging, indicating endothelial cell activation. In addition, TEM imaging showed substantial ultrastructural alterations in LSEC morphology, particularly an increase in damage indicators, as well as an increase in degraded and disrupted cell material caused by detached LSECs, which could be an underlying cause of the increase in permeability observed with SCS. Overall, these findings suggest endothelial activation as a result of SCS, consistent with endothelial dysfunction [62,63,64]. We further confirmed this by showing a significant increase in both immunological and inflammatory cytokine secretion, indicating an injury response to cellular insult [65,66]. Although LSECs appear to withstand SCS better than other hepatic cells, prolonged storage times lead to LSEC dysfunction, resulting in both an inflammatory and immune response.

The changes in LSEC gene expression following SCS further underscore the roles of oxidative stress and apoptosis in preservation-related injury. Interestingly, immediately after 3d SCS, there were virtually no changes in cell death gene expression. This may be attributed to a combination of cold storage that reduces metabolic demand and the components of UW that reduce metabolic activity (high potassium that significantly lowers ATPase activity), mitigate ionic and osmotic stress (potassium, sodium, raffinose, and lactobionic acid), and prevent ROS generation (allopurinol and glutathione), effectively “pausing biological time” [67,68]. Further, while this specific effect has not been demonstrated in LSECs previously, it is documented that endothelial cells in general can survive in hypoxic conditions and provide protective effects to other cells [60,69]. The downregulation of both ROS metabolism and antioxidant genes immediately following SCS suggests that the majority of preservation-related injuries do not occur during cold storage itself. However, once the cells are removed from storage and placed under standard culture conditions, including normoxia, there are drastic changes in gene expression related to ROS metabolism and antioxidants. The upregulation of pro-apoptotic and ROS metabolism genes coupled with the downregulation of antioxidant and oxygen-transport genes indicate substantial oxidative stress. This is in agreement with previous studies that suggest reperfusion is where the majority of LSEC damage occurs during IRI, which subsequently leads to cell death via apoptosis [8,27,28,29,70,71,72].

The therapeutic effects of both CEPT and Trolox have been demonstrated in both ischemic and oxidative stress-damaged cells and tissues [15,73]. CEPT has been shown to promote survival and fitness of human induced pluripotent stem cells, with its components inhibiting cell death through promoting cell survival and growth (chroman 1 and polyamine supplement), pan-caspase inhibition (emricasan), and integrated stress response inhibition (trans-ISRIB) [54,74,75,76]. Additionally, CEPT has shown potential as a therapy for warm ischemic livers [15]. The antioxidant properties of Trolox have been shown to have a therapeutic effect on ischemic hepatic and cardiovascular cells at both the individual cell and total organ level [77,78,79,80,81]. Their beneficial effects here provide further evidence that oxidative stress and apoptosis are significant contributors to preservation-related injury in LSECs. When both apoptosis (inhibited by CEPT) and oxidative stress (inhibited by Trolox) are addressed, 3d SCS + CEPT + Trolox LSECs demonstrate a healthier morphology when compared to 3d SCS without treatment. Viability, metabolic activity, ATP levels, caspase activity, ROS production, Ac-LDL uptake, and heparin scavenging of 3d SCS + CEPT + Trolox LSECs were comparable to, or better than, those of 1d SCS LSECs. Though these improvements in LSEC survival were drastic, the improvements in functionality were markedly less pronounced. It is noteworthy that there was no significant change in permeability or chemokine/cytokine secretion (except for a reduction in EGF secretion) and that morphological indications of damage were still visible in TEM. These observations suggest that while inhibiting both apoptosis and oxidative stress improves LSEC survivability and functionality during SCS, LSECs undergo preservation injury to such an extent that endothelial dysfunction may still occur [62,63,64]. Although loss of function has been noted in LSECs when in a damaging environment, including IRI [8,51], it cannot be ruled out that this could be an artifact of in vitro culture [82,83]. In future studies, we plan to further validate the mechanistic links between CEPT and apoptosis activation and Trolox and oxidative stress pathways. The dynamics of both pathways and how the timing of the treatments affects viability and functional recovery will be of special interest. Additionally, we will also test the effect of these treatments on other hepatic cell types. Of particular interest are hepatocytes, due to their role as the main functional cell type of the liver, and the biliary epithelium due to its critical role in bile composition and distinct susceptibility to preservation injury, particularly in SCS [3,30,31,59,78]. Further, we will explore the role of various other treatments to promote better LSEC functionality and recovery following SCS to further extend preservation time. One such mechanism of interest is the effect of shear stress, which has been shown to play a role in endothelial dysfunction, particularly through transcription factor Klf2 expression [51,84].

These findings suggest that, although inhibition of apoptosis and oxidative stress improves LSEC survival, significant cellular stress persists, which may impair their ability to maintain protective functions after prolonged preservation [8,29,60]. Furthermore, these results provide insight into the role of LSECs in shaping the overall hepatic response to IRI injury. Increased ROS production indicates reduced redox capacity, while increased membrane permeability suggests compromised endothelial integrity. At the organ level, these changes may impair microvascular function and oxygen exchange, thereby exacerbating IRI in hepatocytes and contributing to reduced liver function. Additionally, increased heparin scavenging, Ac-LDL uptake, and elevated chemokine and cytokine secretion suggest that injured LSECs promote an inflammatory response. In the whole liver, this inflammatory response may further damage hepatocytes and worsen functional outcomes, particularly in marginal livers. Notably, the observed changes in chemokine and cytokine secretion also highlight their potential as therapeutic targets and/or biomarkers for IRI and graft assessment. However, it must be noted that this study is on isolated LSECs in vitro and that the extrapolation of these results must be further validated in more complex models. Our studies have shown the beneficial effects of both CEPT and its components in rescued whole livers and hypothesize that the addition of antioxidants would only be beneficial [15,85,86]. However, to address this and definitively prove the beneficial effects in a more complex system, future studies will evaluate whether these findings translate to both co-culture and whole-liver systems, including machine perfusion models, to better define the interplay between LSEC preservation injury and overall liver function.

Though promising, the results from this initial study come with several limitations due to the nature of using a primary isolated cell in vitro model. This includes that though our model includes cold ischemia and subsequent warm and sudden reoxygenation, it does not exactly replicate clinical IRI seen in liver transplant, particularly due to the lack of immune cells within the model. Further, though the isolation protocol used here leads to >90% purity, the presence and effect of other cell types (particularly KCs) cannot be ruled out. On a similar note, the goal of this study was to better understand the effect of preservation injury in LSECs specifically; the damage and activation of other hepatic cell types may have a drastic effect on LSEC viability and functionality that is not observed here. Additionally, in future studies, we plan to perform additional experiments with our current in vitro model, both as controls and to increase the statistical power and confirm the reproducibility of our results, particularly for our experiments with lower biological replicates. Nevertheless, the results from this study, along with prior work from both our lab [15,85] and others [73,77,78,79,80,81], suggest that CEPT and Trolox represent promising candidates for use beyond SCS, particularly in both machine-perfusion and extended storage methods (such as subzero storage) for liver transplant. While these strategies have been shown to significantly extend organ storage time, they remain limited by persistent IRI [10,11,14,19,20,21]. Incorporating apoptosis and oxidative stress inhibitors into storage and/or perfusion solutions may further mitigate endothelial injury, improve microvascular function during reperfusion, and enhance overall graft recovery. Such approaches could increase the utilization of marginal organs by improving endothelial resilience and preserving organ quality during extended storage.

## 5. Conclusions

In conclusion, this study demonstrates that the timeline of preservation injury in LSECs differs from that of other hepatic cell types, including hepatocytes, HSCs, and KCs. Our findings identify oxidative stress during recovery as a significant driver of apoptosis in LSECS following SCS. While inhibition of apoptosis with CEPT and oxidative stress with Trolox partially improves LSEC viability, functional recovery remains incomplete. These results highlight the importance of understanding LSEC-specific injury mechanisms and provide a foundation for the development of novel endothelial cell-targeted preservation strategies to improve liver transplantation outcomes, with potential relevance to other solid organs.

## Data Availability

The raw data supporting the conclusions of this article will be made available by the authors on request.

## Supplementary Materials

The following supporting information can be downloaded at: https://www.mdpi.com/article/10.3390/cells15151334/s1, Figure S1: LSEC secreted supernatant levels of cytokines and chemokines (A) IFN-g, (B) IL-5, (C) IL-6, (D) IL-10, (E) IL-13, (F) IL-18, (G) LIX, and (H)MCP-1 in 1d or 3d SCS. Sample size: *n* = 3 for all. Figure S2: Effect of heat shock and PEG on LSECs following 3 days of SCS. (A) Metabolic activity (B) ATP levels and (C) caspase activity of LSECs following 3d SCS with either no treatment, heat shock, PEG, or heat shock + PEG. Samples size: (A) 3d SCS *n* = 16, (B) 3d SCS *n* =15, (C) 3d SCS *n* = 15, for all other groups *n* = 2. Figure S3: Effect of CEPT and Trolox on LSECs following 3 days of SCS and 24 h of recovery. (A) Representative Live/Dead images of LSECs following 3d SCS and 24 h of recovery either with no treatment, CEPT, Trolox, or CEPT + Trolox treatment. (B) Viability, (C) metabolic activity, (D) ATP levels, (E) caspase activity, and (F) ROS production of LSECs following 3d SCS and 24 h of recovery either with no treatment, CEPT, Trolox, or CEPT + Trolox treatment. Scale Bar 300 µm. * denotes p < 0.05. Sample size: (B) No Treatment *n* = 9, CEPT *n* = 6, Trolox *n* = 5, CEPT + Trolox *n* = 7, (C) No Treatment *n* = 15, CEPT *n* = 6, Trolox *n* = 5, CEPT + Trolox *n* = 8, (D) No Treatment *n* = 15, CEPT *n* = 5, Trolox *n* = 7, CEPT + Trolox *n* = 5, (E) No Treatment *n* = 15, CEPT *n* = 3, Trolox *n* = 2, CEPT + Trolox *n* = 7, (F) *n* = 3 for all. Figure S4: CEPT and Trolox treatment had little effect on LSEC secretion of cytokines effected by SCS. LSEC secreted supernatant levels of cytokines and chemokines (A) IL-1a, (B) MIP-1a, (C) TNF-a, (D) IL-1b, (E) MIP-2, (F) IP-10, (G) IL-4, (H) GRO, (I) Leptin, (J) RANTES, or (K) VEGF following 3d SCS either with or without CEPT + Trolox. Sample Size: *n* = 3 for all. Figure S5: Effect of CEPT and Trolox treatment on LSEC cytokine expression. LSEC secreted supernatant levels of cytokines (A) IFN- g, (B) IL-5, (C) IL-6, (D) IL-10, (E) IL-13, (F) IL-18, (G) LIX, and (H) MCP-1 following 3d SCS either with or without CEPT + Trolox. Sample Size: *n* = 3 for all. Table S1: Cell Death Related Gene Expression Following 1 or 2 Days of Static Cold Storage (SCS) and Either 0 or 24 Hours of Recovery. Table S2: Oxidative Stress Related Gene Expression Following 1 or 2 Days of Static Cold Storage (SCS) and Either 0 or 24 Hours of Recovery.
