# Supplementary material for: Oxidative Stress and Apoptosis Inhibition Mitigate Static Cold Storage-Induced Injury in Liver Sinusoidal Endothelial Cells
Source: Cells. 2026 Jul 25;15(15):1334. doi: 10.3390/cells15151334 (PMC13464770; doi:10.3390/cells15151334)
Supplement: Supplementary file 1 [file cells-15-01334-s001.zip › cells-4403126-supplementary.pdf]

Supplemental Material

- **Table of Contents** pg. 1
- **Supplemental Figure S1** pg. 2
- **Supplemental Figure S2** pg. 3
- **Supplemental Figure S3** pg. 4
- **Supplemental Figure S4** pg. 5
- **Supplemental Figure S5** pg. 6
- **Supplemental Table S1** pg. 7
- **Supplemental Table S2** pg. 8

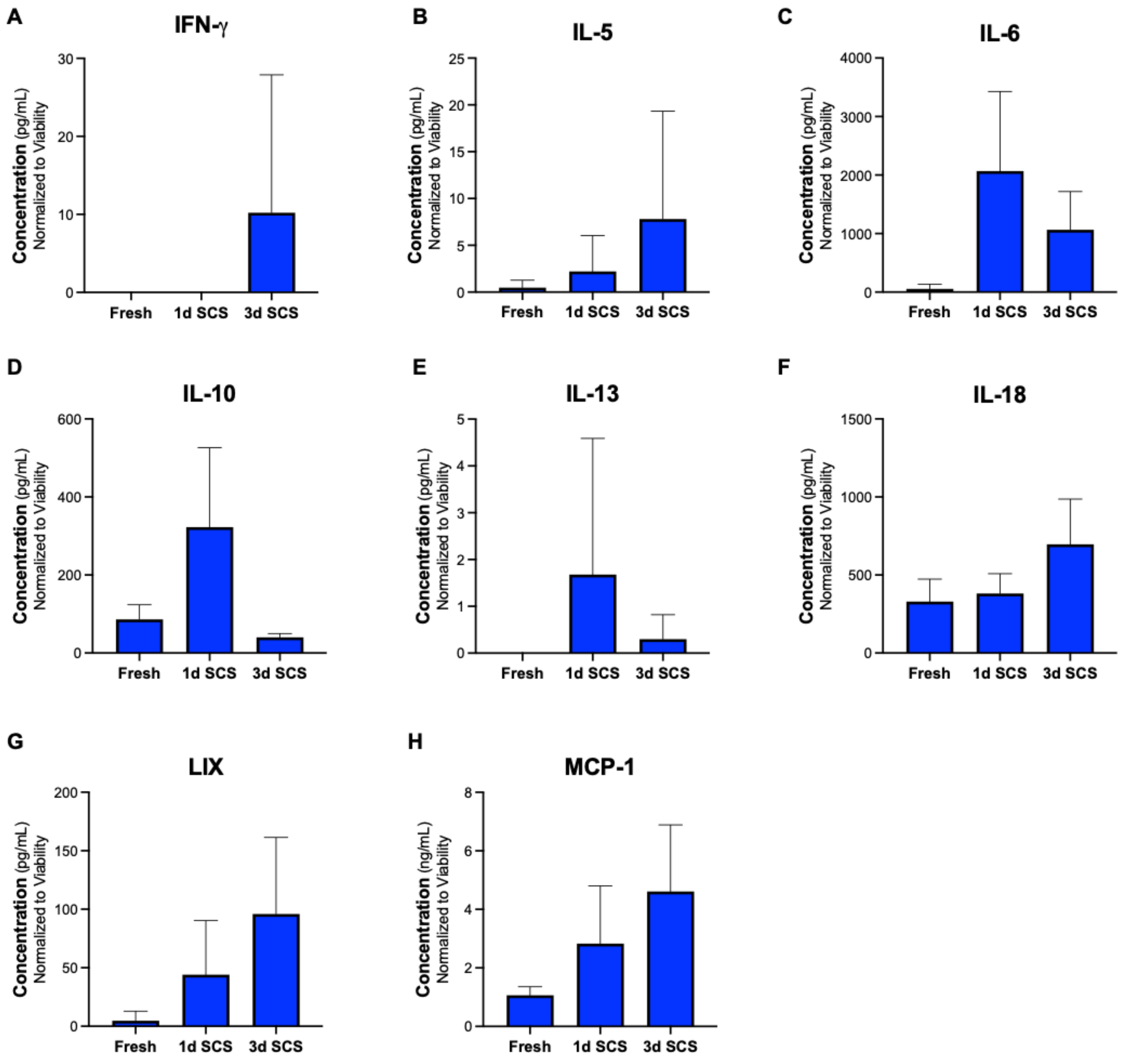

Supplemental Figure S1. Effect of SCS on LSEC cytokine expression. LSEC secreted supernatant levels of cytokines and chemokines **(A)** IFN-γ, **(B)** IL-5, **(C)** IL-6, **(D)** IL-10, **(E)** IL-13, **(F)** IL-18, **(G)** LIX, and **(H)** MCP-1 in 1d or 3d SCS. Sample size: n = 3 for all.

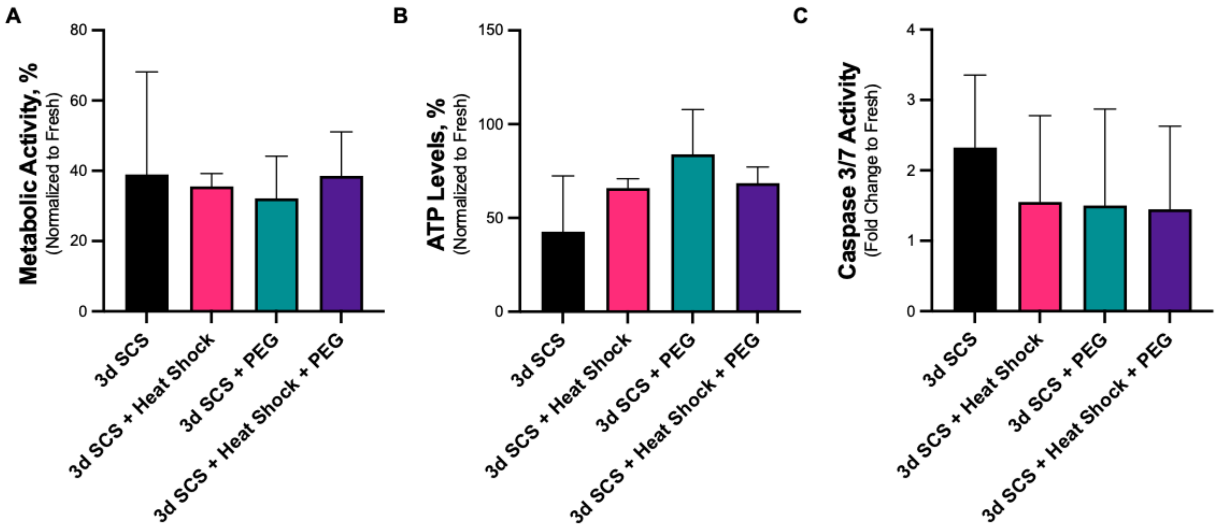

Supplemental Figure S 2. Effect of heat shock and PEG on LSECs following 3 days of SCS. (A) Metabolic activity **(B)** ATP levels and **(C)** caspase activity of LSECs following 3d SCS with either no treatment, heat shock, PEG, or heat shock + PEG. Samples size: (A) 3d SCS n = 16, (B) 3d SCS n =15, (C) 3d SCS n = 15, for all other groups n = 2.

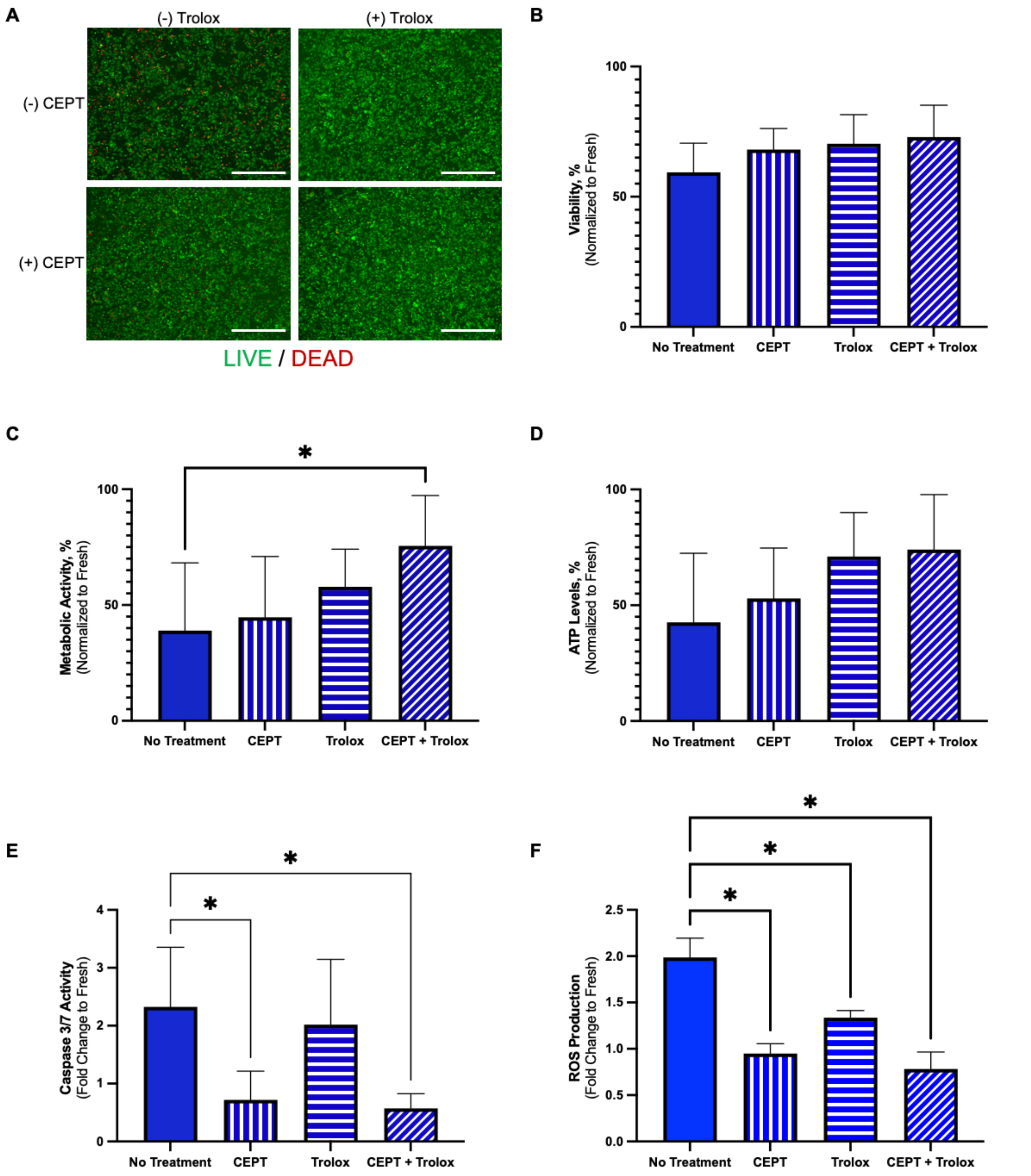

Supplemental Figure S3. Effect of CEPT and Trolox on LSECs following 3 days of SCS and 24 h of recovery. (A) Representative Live/Dead images of LSECs following 3d SCS and 24 h of recovery either with no treatment, CEPT, Trolox, or CEPT + Trolox treatment. **(B)** Viability, **(C)** metabolic activity, **(D)** ATP levels, **(E)** caspase activity, and **(F)** ROS production of LSECs following 3d SCS and 24 h of recovery either with no treatment, CEPT, Trolox, or CEPT + Trolox treatment. Scale Bar 300 μm. * denotes $p < 0.05$. Sample size: (B) No Treatment n = 9, CEPT n = 6, Trolox n = 5, CEPT + Trolox n = 7, (C) No Treatment n = 15, CEPT n = 6, Trolox n = 5, CEPT + Trolox n = 8, (D) No Treatment n = 15, CEPT n = 5, Trolox n = 7, CEPT + Trolox n = 5, (E) No Treatment n = 15, CEPT n = 3, Trolox n = 2, CEPT + Trolox n = 7, (F) n = 3 for all.

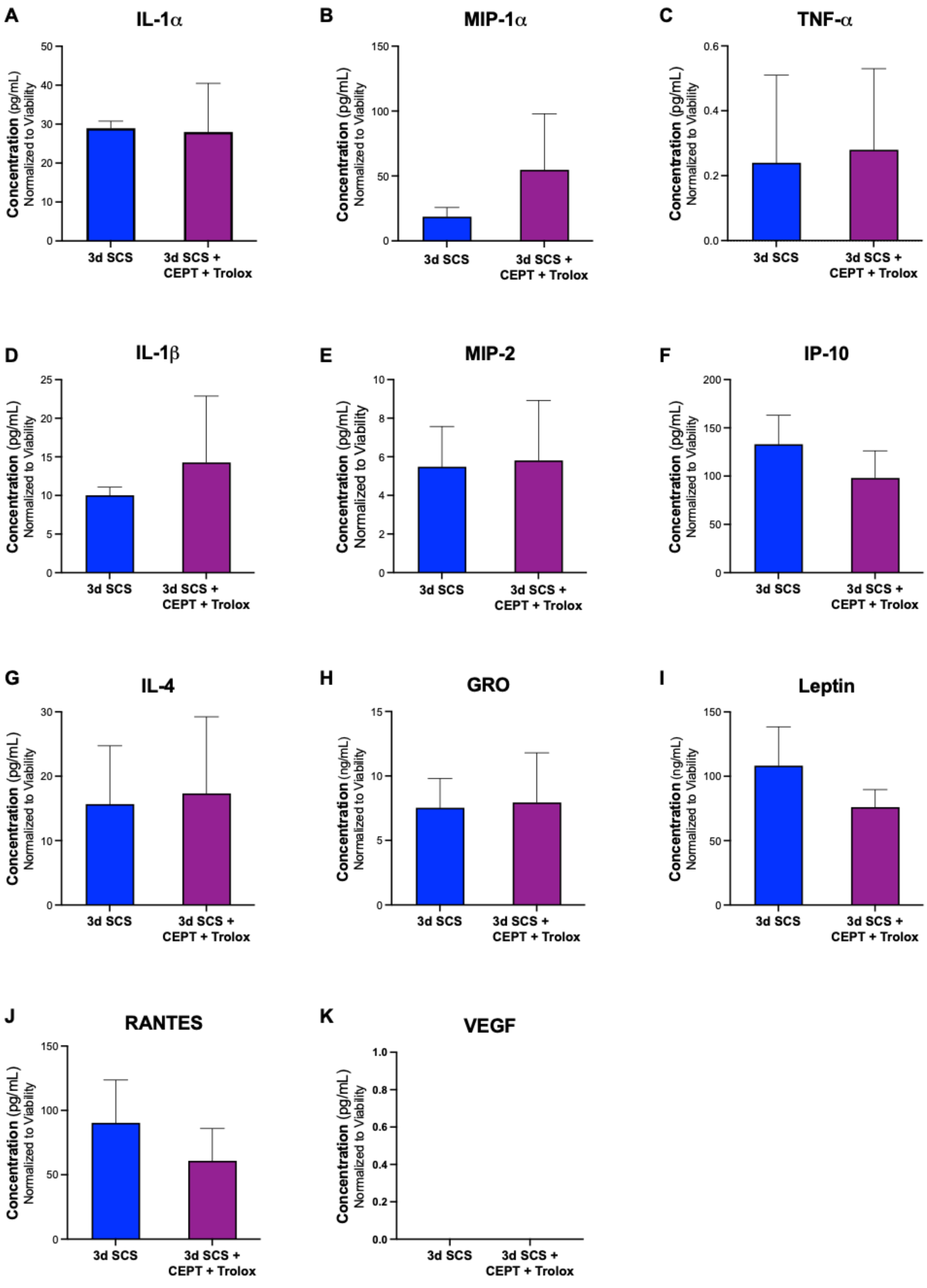

Supplemental Figure S4. CEPT and Trolox treatment had little effect on LSEC secretion of cytokines effected by SCS. LSEC secreted supernatant levels of cytokines and chemokines **(A)** IL-1α, **(B)** MIP-1α, **(C)** TNF-α, **(D)** IL-1β, **(E)** MIP-2, **(F)** IP-10, **(G)** IL-4, **(H)** GRO, **(I)** Leptin, **(J)** RANTES, or **(K)** VEGF following 3d SCS either with or without CEPT + Trolox. Sample Size: n = 3 for all

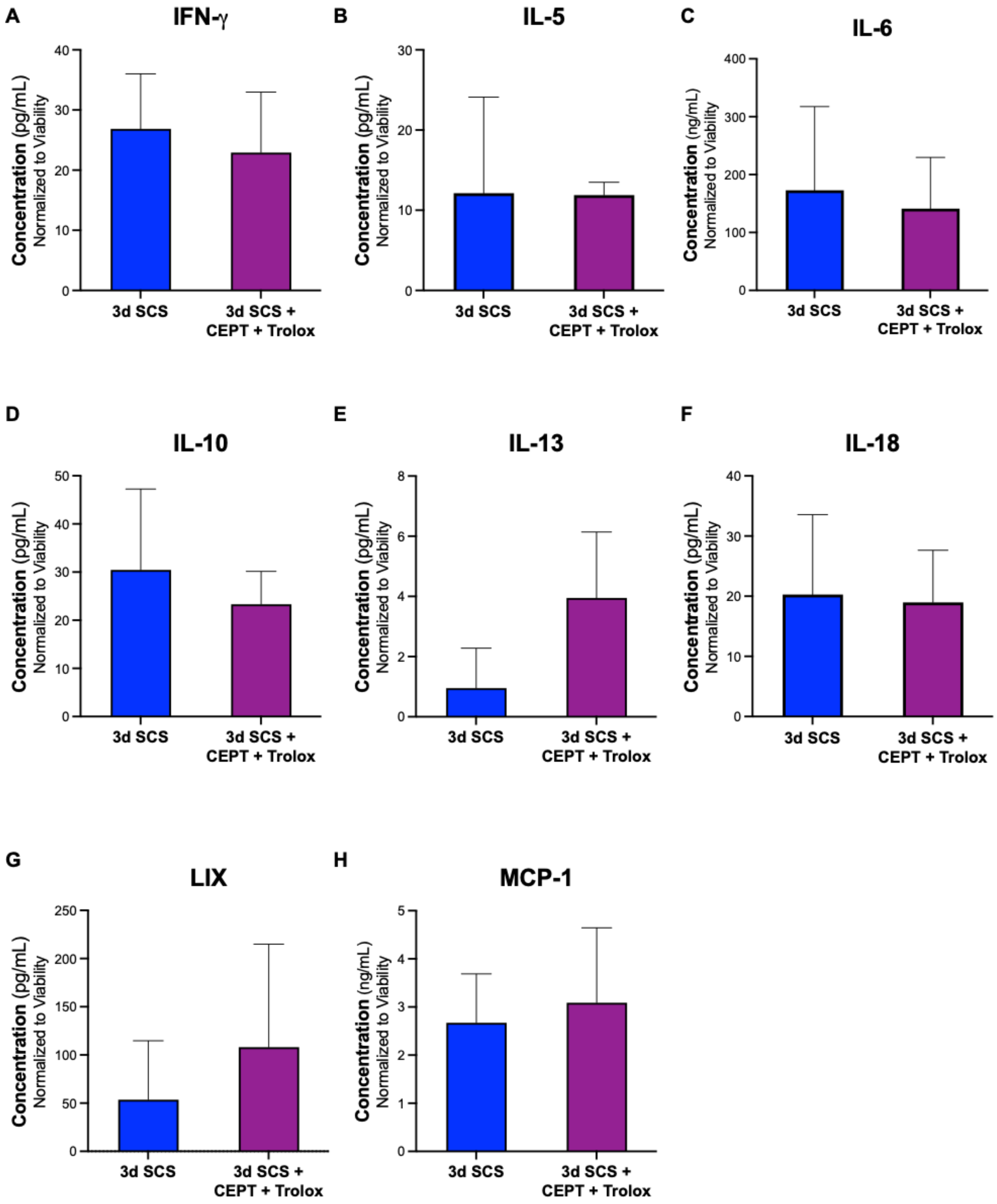

Supplemental Figure S5. Effect of CEPT and Trolox treatment on LSEC cytokine expression. LSEC secreted supernatant levels of cytokines **(A)** IFN-γ, **(B)** IL-5, **(C)** IL-6, **(D)** IL-10, **(E)** IL-13, **(F)** IL-18, **(G)** LIX, and **(H)** MCP-1 following 3d SCS either with or without CEPT + Trolox. Sample Size: n = 3 for all

Supplemental Table S1: Cell Death Related Gene Expression Following 1 or 2 Days of Static Cold Storage (SCS) and Either 0 or 24 Hours of Recovery

Gene			Fold Change to Fresh (* = p < 0.05)				
Symbol	Name	Role	Fresh	1D SCS; 0hr Recovery	1D SCS; 24hr Recovery	3D SCS; 0hr Recovery	3D SCS; 24hr Recovery
Abl1	ABL Proto-Oncogene 1	Pro-Apoptosis	1.074	0.663	0.858	0.827	2.080*
Akt1	AKT Serine/Threonine Kinase 1	Anti-Apoptosis, Autophagy	1.068	0.537	0.505	0.608	1.048
Apaf1	Apoptotic Peptidase Activating Factor 1	Pro-Apoptosis	1.025	0.777	0.713	0.733	2.154*
App	Amyloid Beta Precursor Protein	Autophagy	1.058	0.739	0.540	0.527	0.980
Atg12	Autophagy Related 12	Autophagy	1.033	1.143	1.250	0.893	1.873
Atg16l1	Autophagy Related 16 like 1	Autophagy	1.041	0.864	1.060	0.965	1.704
Atg3	Autophagy Related 3	Autophagy	1.049	0.866	1.174	0.591	1.371
Atg5	Autophagy Related 5	Autophagy	1.014	0.956	0.790	0.814	1.319
Atg7	Autophagy Related 7	Autophagy	1.120	0.656	0.598	0.540	0.811
Atp6v1g2	ATPase H+ Transporting V1 Subunit G2	Pro-Apoptosis, Necrosis	1.041	0.671	2.490*	0.722	1.627
Bax	BCL2 Associated X	Pro-Apoptosis, Autophagy	1.056	1.618	1.639	1.376	3.248*
Bcl2	BCL2 Apoptosis Regulator	Anti-Apoptosis, Autophagy	1.011	0.607	0.694	0.651	0.715
Bcl2a1	BCL2 Related Protein A1	Anti-Apoptosis	1.009	1.292	0.929	1.036	0.085*
Bcl2l1	BCL2 Like 1	Anti-Apoptosis, Autophagy	1.039	1.900	1.152	1.428	2.444*
Bcl2l11	BCL2 Like 11	Pro-Apoptosis	1.044	0.738	0.845	0.539	1.901
Becn1	Beclin 1	Autophagy	1.056	0.675	1.346	0.871	2.376*
Birc2	Baculoviral IAP Repeat Containing 2	Pro-Apoptosis	1.031	1.342	0.724	0.651	1.179
Birc3	Baculoviral IAP Repeat Containing 3	Anti-Apoptosis	1.003	1.686	1.313	1.192	3.181*
Bmf	Bcl2 Modifying Factor	Necrosis	1.074	0.424	0.227*	0.385	0.516
Casp1	Caspase 1	Pro-Apoptosis	1.060	0.654	0.590	0.470	0.540
Casp2	Caspase 2	Anti-Apoptosis	1.078	0.496	0.461	0.492	1.294
Casp3	Caspase 3	Pro-Apoptosis, Autophagy	1.027	1.011	0.910	0.765	2.239*
Casp6	Caspase 6	Pro-Apoptosis	1.021	0.437*	0.939	0.650	1.105
Casp7	Caspase 7	Pro-Apoptosis	1.057	0.814	0.777	0.734	1.420
Casp9	Caspase 9	Pro-Apoptosis	21.274	12.883	14.752	18.932	19.297
Cd40	CD40 Molecule	Pro-Apoptosis	1.028	1.845	1.077	1.121	1.988
Cd40lg	CD40 Ligand	Pro-Apoptosis	3.732	11.887	9.325	6.877	0.041
Cflar	CASP8 And FAFF Like Apoptosis Regulator	Pro-Apoptosis	1.087	2.059	1.332	1.121	2.781*
Commd4	COMM Domain Containing 4	Necrosis	1.076	1.023	1.119	1.096	1.382
Ctsb	Cathepsin B	Autophagy	1.084	0.817	0.455	0.552	0.543
Ctss	Cathepsin S	Autophagy	1.103	0.251	0.158*	0.180*	0.010*
Cybb	Cytochrome B-245 Beta Chain	Necrosis	1.038	0.341*	0.164*	0.355	0.026*
Cyld	CYLD lysine 63 Deubiquitinase	Pro-Apoptosis, Necrosis	1.026	0.482	0.454*	0.568	0.716
Defb1	Defensin Beta 1	Necrosis	1.153	0.786	4.175	4.869	0.238
Dffa	DNA Fragmentation Factor Subunit Alpha	Pro-Apoptosis	1.120	0.954	0.999	0.777	1.507
Dpysl4	Dihydropyrimidinase Like 4	Necrosis	4.606	22.012	27.401	25.389	107.385*
Esr1	Estrogen Receptor 1	Autophagy	1.097	0.219	0.135*	0.925	0.171
Fas	Fas Cell Surface Death Receptor	Pro-Apoptosis, Autophagy	1.006	0.887	0.767	1.642	1.819
Faslg	Fas Ligand	Pro-Apoptosis	1.258	7.022	6.890	8.060	2.510
Foxi1	Forkhead Box I1	Necrosis	1.285	1.188	2.260	5.279	10.611
Gaa	Alpha Glucosidase	Autophagy	1.060	0.662	0.363*	0.510	0.491
Gadd45a	Growth Arrest and DNA Damage Inducible Alpha	Pro-Apoptosis	1.047	2.317	4.022	1.430	4.108*
Galnt5	Polypeptide N-Acetylgalactosaminyltransferase 5	Necrosis	1.383	0.765	0.825	0.659	0.383
Grb2	Growth Factor Receptor Bound Protein 2	Necrosis	1.039	0.659	0.544	0.567	0.718
Hspbap1	HSPB1 Associated Protein 1	Necrosis	1.033	0.817	1.846	0.921	1.598
Htt	Huntingtin	Autophagy	1.079	0.558	0.475	0.539	1.060
Ifng	LFNG O-Fucosylpeptide 3-Beta-N-Acetylglucosaminyltransferase	Autophagy	6.261	5.713	4.970	18.082	13.049
Igf1	Insulin Like Growth Factor 1	Autophagy	1.089	0.420	0.644	0.494	0.040*
Igf1r	Insulin Like Growth Factor 1 Receptor	Anti-Apoptosis	1.182	0.742	0.594	0.464	1.166
Ins2	Insulin	Autophagy	12.602	1.224	86.473	151.499	416.202
Irgm	Immunity Related GTPase M	Autophagy	1.015	0.560	0.532	0.798	0.405*
Kcnip1	Potassium Voltage-Gated Channel Interacting Protein 1	Necrosis	1.047	1.289	1.326	1.871	1.320
Mag	Myelin Associated Glycoprotein	Necrosis	1.019	17.779	1.488	31.867	1.482
Map1lc3a	Microtubule Associated Protein 1 Light Chain 3 Alpha	Autophagy	1.014	0.797	0.785	0.718	0.828
Mapk8	Mitogen-Activated Protein Kinase 8	Autophagy	1.084	0.796	0.877	0.440	1.401
Mcl1	MCL1 Apoptosis Regulator, BCL2 Family Member	Anti-Apoptosis	1.035	0.824	0.789	0.696	1.265
Nfkb1	Nuclear Factor Kappa B Subunit 1	Autophagy	1.022	0.960	0.688	0.624	2.251*
Nol3	Nucleolar Protein 3	Pro-Apoptosis	1.048	1.686	2.265	0.991	2.593*
Olr1583	Olfactory receptor 1583	Necrosis	1.151	0.249	1.509	0.665	1.432
Parp1	Poly(ADP-Ribose) Polymerase 1	Necrosis	1.034	0.416	0.394*	0.467	0.699
Parp2	Poly(ADP-Ribose) Polymerase 2	Necrosis	1.044	0.491	0.770	0.695	1.360
Pik3c3	Phosphatidylinositol 3-Kinase Catalytic Subunit Type 3	Autophagy	1.050	0.671	0.693	0.694	1.216
Pten	Phosphatase and Tensin Homolog	Autophagy	1.060	0.608	0.811	0.487	1.145
Pvr	PVR Cell Adhesion Molecule	Necrosis	1.081	2.501	1.817	1.649	3.236*
Rab25	Member RAS Oncogene Family	Necrosis	1.023	0.693	0.837	0.808	0.973
RGD1311517	N-acetylglucosamine-1-phosphate transferase	Necrosis	1.013	0.874	1.320	1.199	2.066*
Dennd4a	DENN Domain Containing 4A	Necrosis	1.012	1.072	0.823	0.808	0.824
Rps6kb1	Ribosomal Protein S6 Kinase B1	Autophagy	1.044	0.909	1.015	0.650	1.733
Snca	Synuclein Alpha	Autophagy	4.542	0.234	6.514	17.133	6.530
Spata2	Spermatogenesis Associated 2	Pro-Apoptosis, Necrosis	1.113	0.492	0.459	0.568	0.934
Sqstm1	Sequestosome 1	Autophagy	1.070	1.860	1.684	1.093	1.199
Sycp2	Synaptonemal Complex Protein 2	Pro-Apoptosis, Necrosis	1.255	2.441	1.900	0.978	1.631
Maco1	Macoilin 1	Necrosis	1.055	1.048	0.747	0.489	1.282
Tnf	Tumor Necrosis Factor	Pro-Apoptosis, Autophagy	1.025	3.855	0.729	1.663	0.608
Tnfrsf10b	TNF Receptor Superfamily Member 10b	Pro-Apoptosis	1.032	0.760	1.143	0.916	2.411*
Tnfrsf11b	TNF Receptor Superfamily Member 11b	Anti-Apoptosis	1.759	0.031	0.017	0.054	0.416
Tnfrsf1a	TNF Receptor Superfamily Member 1a	Pro-Apoptosis, Necrosis	1.064	0.976	0.511	0.685	1.093
Tnfrsf4	TNF Receptor Superfamily Member 4	Necrosis	9.333	9.880	11.522	12.464	6.089
Tnfrsf8	TNF Receptor Superfamily Member 8	Necrosis	1.038	0.068*	0.102*	0.204*	0.065*
Tp53	Tumor Protein P53	Pro-Apoptosis, Autophagy	1.014	1.943	1.545	1.329	2.661*
Traf2	TNF Receptor Associated Factor 2	Anti-Apoptosis	1.021	1.310	1.154	1.071	2.342*
Txnl4b	Thioredoxin Like 4B	Necrosis	1.064	0.827	0.941	0.808	1.355
Ulk1	Unc-51 Like Autophagy activating Kinase 1	Autophagy	1.039	0.556	0.603	0.545	1.042
Xiap	X-Linked Inhibitor of Apoptosis	Anti-Apoptosis	1.033	0.886	0.720	0.678	1.666

Supplemental Table S2: Oxidative Stress Related Gene Expression Following 1 or 2 Days of Static Cold Storage (SCS) and Either 0 or 24 Hours of Recovery

Gene			Fold Change (* = p < 0.05)				
Symbol	Name	Role	Fresh	1D SCS; 0hr Recovery	1D SCS; 24hr Recovery	3D SCS; 0hr Recovery	3D SCS; 24hr Recovery
Alb	Albumin	Other Antioxidants	3.246	2.761	0.728	4.171	0.306
Als2	Amyotrophic Lateral Sclerosis Type 2	Oxidative Stress Responsive	0.952	0.926	1.586	0.742	1.704
Aox1	Aldehyde Oxidase 1	Other ROS Metabolism	1.850	0.287	0.775	1.133	0.343
Apc	APC Regulator of WNT Signaling Pathway	Other Peroxidases	0.922	0.701	1.619	0.511	1.769
Apoe	Apolipoprotein E	Oxidative Stress Responsive	1.172	0.561	0.285*	0.964	0.316*
Cat	Catalase	Oxidative Stress Responsive, Other Peroxidases	1.090	0.632	0.657	1.109	0.326*
Ccl5	C-C Motif Chemokine Ligand 5	Oxidative Stress Responsive	6.291	22.749	1.545	92.749	0.925
Ccs	Copper Chaperone for Superoxide Dismutase	ROS SOD (Other Genes)	1.015	0.567	1.000	0.479*	0.860
Ctsb	Cathepsin B	Oxidative Stress Responsive, Other Peroxidases	0.977	0.633	0.597	0.667	0.468*
Cyba	Cytochrome B-245 Alpha Chain	ROS SOD (Other Genes)	0.956	0.537	0.372*	0.726	0.124*
Cygb	Cytochrome B-245 Beta Chain	Oxygen Transporters	1.064	0.194	0.044*	0.116	0.130
Dhcr24	24-Dehydrocholesterol Reductase	Oxidative Stress Responsive	1.919	0.098	0.294	0.401	0.207
Dnm2	Dynamin 2	Oxygen Transporters	0.943	0.596	0.872	0.535	0.950
Duox1	Dual Oxidase 1	Oxidative Stress Responsive, Other Peroxidases	1.179	1.371	1.585	1.806	2.396
Duox2	Dual Oxidase 2	Oxidative Stress Responsive	1.321	0.625	0.703	1.777	0.830
Ehd2	EH Domain Containing 2	Antioxidant (TPx)	0.840	0.051*	0.018*	0.034*	0.436
Epx	Eosinophil Peroxidase	Oxidative Stress Responsive, Other Peroxidases	1.201	0.350	2.553	1.164	0.711
Ercc2	ERCC Excision Repair 2	Oxidative Stress Responsive	0.972	0.740	1.580	1.048	2.003
Ercc6	ERCC Excision Repair 6	Oxidative Stress Responsive	1.063	0.732	2.162	0.660	2.103
Fancc	FA Complementation Group C	Oxygen Transporters	1.002	0.595	1.600	0.678	1.612
Fmo2	Flavin Containing Dimethylaniline Monooxygenase 2	Other ROS Metabolism	5.970	1.121	232.064	0.576	1.712
Fth1	Ferritin Heavy Chain 1	Oxidative Stress Responsive	0.909	0.593	1.125	0.825	0.232*
Gclc	Glutamate-Cysteine Ligase Catalytic Subunit	Oxidative Stress Responsive	0.958	0.800	2.522	0.859	1.138
Gclm	Glutamate-Cysteine Ligase Modifier Subunit	Oxidative Stress Responsive	0.989	0.861	2.309	0.849	0.845
Gpx1	Glutathione Peroxidase 1	Oxidative Stress Responsive, Antioxidant (GPx)	0.991	0.878	0.707	1.025	0.509
Gpx2	Glutathione Peroxidase 2	Oxidative Stress Responsive, Antioxidant (GPx)	2.029	0.261	0.994	1.352	0.536
Gpx3	Glutathione Peroxidase 3	Oxidative Stress Responsive, Antioxidant (GPx)	0.956	0.067	0.098	0.033	0.164
Gpx4	Glutathione Peroxidase 4	Oxidative Stress Responsive, Antioxidant (GPx)	0.992	0.900	1.007	1.088	1.234
Gpx5	Glutathione Peroxidase 5	Oxidative Stress Responsive, Antioxidant (GPx)	2.713	1.290	4.774	1.518	7.001
Gpx6	Glutathione Peroxidase 6	Oxidative Stress Responsive, Antioxidant (GPx)	0.915	1.125	1.189	44.719	2.996
Gpx7	Glutathione Peroxidase 7	Oxidative Stress Responsive, Antioxidant (GPx)	0.861	0.194*	0.548	0.145*	1.214
Gsr	Glutathione-Disulfide Reductase	Oxidative Stress Responsive, Other Antioxidants	1.040	0.716	0.921	0.938	0.560
Gstk1	Glutathione S-Transferase Kappa 1	Antioxidant (GPx)	1.184	0.672	0.600	0.912	0.742
Gstp1	Glutathione S-Transferase Pi 1	Antioxidant (GPx)	0.939	1.362	1.054	1.204	1.770
Hba-a1	Hemoglobin Subunit Alpha 1	Oxygen Transporters	1.695	3.951	4.689	12.538	2.605
Hmox1	Heme Oxygenase 1	Oxidative Stress Responsive	0.892	0.596	2.085	0.748	0.263*
Hspa1a	Heat Shock Protein Family A (Hsp70) Member 1A	Oxidative Stress Responsive	2.882	2.936	111.561	2.717	11.462*
Idh1	Isocitrate Dehydrogenase (NADP(+))1	Oxidative Stress Responsive	1.016	0.450*	0.557	0.435*	0.385*
Krt1	Keratin 1	Oxidative Stress Responsive	1.000	0.225*	1.814	0.295*	0.967
LOC367198		Oxygen Transporters	1.539	0.352	2.940	0.819	0.578
Lpo	Lactoperoxidase	Other Peroxidases	1.087	0.228*	0.122*	0.664	0.073*
Mb	Myoglobin	Oxygen Transporters	0.912	65.108	304.498	195.342	3.326
Mpo	Myeloperoxidase	Oxidative Stress Responsive, Other Peroxidases	11.134	0.616	2.976	4.190	0.394
Ncf1	Neutrophil Cytosolic Factor 1	ROS SOD (Other Genes)	1.041	0.643	0.276*	0.931	0.039*
Ncf2	Neutrophil Cytosolic Factor 2	ROS SOD (Other Genes)	0.966	0.639	0.672	0.905	0.087*
Ngb	Neuroglobin	Oxygen Transporters	4.213	11.915	53.063	32.061	16.878
Nos2	Nitric Oxide Synthase 2	ROS SOD (Other Genes)	1.834	0.986	0.415	6.605	2.299
Nox4	NADPH Oxidase 4	ROS SOD (Other Genes)	0.817	0.060	0.171	0.103	0.144
Noxa1	NADPH Oxidase Activator 1	ROS SOD (Other Genes)	0.772	0.356	1.254	0.278	0.218
Noxo1	NADPH Oxidase Organizer 1	ROS SOD (Other Genes)	3.002	4.147	13.516*	5.937	4.518
Nqo1	NAD(P)H Quinone Dehydrogenase 1	Oxidative Stress Responsive	0.944	3.197*	4.020	2.950*	3.491*
Nudt1	Nudix Hydrolase 1	Oxidative Stress Responsive	1.011	0.683	1.293	0.575	2.206*
Park7	Parkinsonism Associated Deglycase	Oxidative Stress Responsive	1.022	0.689	0.837	0.723	1.594
Prdx1	Peroxiredoxin 1	Oxidative Stress Responsive, Antioxidant (TPx)	0.963	0.942	1.281	0.763	0.883
Prdx2	Peroxiredoxin 2	Oxidative Stress Responsive, Antioxidant (TPx)	0.973	0.873	1.048	1.031	1.340
Prdx3	Peroxiredoxin 3	Antioxidant (TPx)	1.091	0.824	0.956	0.876	0.907
Prdx4	Peroxiredoxin 4	Antioxidant (TPx)	0.962	0.601	0.616	0.647	1.062
Prdx5	Peroxiredoxin 5	Antioxidant (TPx)	1.045	1.454	1.167	1.680	0.547
Prdx6	Peroxiredoxin 6	Oxidative Stress Responsive, Antioxidant (TPx)	0.927	1.282	1.850	1.072	1.634
Prnp	Prion Protein	Oxidative Stress Responsive	0.870	0.842	1.682	0.562	1.518
Psmb5	Proteasome 20S Subunit Beta 5	Oxidative Stress Responsive	1.041	1.239	1.089	1.059	1.597
Ptgs1	Prostaglandin-Endoperoxide Synthase 1	Other Peroxidases	1.089	0.648	0.307*	0.744	0.056*
Ptgs2	Prostaglandin-Endoperoxide Synthase 2	Other Peroxidases	0.983	14.425	14.063	8.119	10.020*
Rag2	Recombination Activating 2	Other Peroxidases	2.876	12.609	0.439	28.430	7.337
Tpo	Thyroid Peroxidase	Oxidative Stress Responsive	0.912	0.773	1.413	1.797	1.268
Txn1	Thioredoxin	Oxidative Stress Responsive	0.992	1.395	1.818	1.516	1.204
Txnrd1	Thioredoxin Reductase 1	Oxidative Stress Responsive, Other Antioxidants	0.924	1.409	2.241	1.257	1.107
Txnrd2	Thioredoxin Reductase 2	Oxidative Stress Responsive, Other Antioxidants	0.958	0.555	1.047	0.788	1.149
Scd	Stearoyl-CoA Desaturase	ROS SOD (Other Genes)	3.531	3.575	0.937	5.545	0.957
Selenop	Selenoprotein P	Oxidative Stress Responsive	1.016	0.182*	0.197*	0.083*	0.152*
Selenos	Selenoprotein S	Oxidative Stress Responsive, Other Antioxidants	0.955	0.849	1.449	0.852	0.909
Serpinb1b	Serpin Family B Member 1	Other Peroxidases	1.064	0.194	0.044*	0.116	0.130*
Slc38a1	Solute Carrier Family 38 Member 1	Oxygen Transporters	1.196	1.789	1.469	1.065	2.277
Slc38a5	Solute Carrier Family 38 Member 5	Oxygen Transporters	3.415	3.136	6.044	34.961	20.359
Sod1	Superoxide Dismutase 1	Oxidative Stress Responsive, ROS SOD, Other Antioxidants	0.985	1.014	1.564	0.887	1.097
Sod2	Superoxide Dismutase 2	ROS SOD	1.134	2.521*	1.742	2.697	4.566*
Sod3	Superoxide Dismutase 3	ROS SOD, Other Antioxidants	1.087	0.642	0.792	0.285	0.407
Sqstm1	Sequestosome 1	Oxidative Stress Responsive	0.997	1.711	2.955	1.468	1.324
Srxn1	Sulfiredoxin 1	Other Antioxidants	0.909	2.766	8.365	3.007*	3.220*
Ucp2	Uncoupling Protein 2	ROS SOD (Other Genes)	1.130	0.443	0.418	0.547	0.673
Ucp3	Uncoupling Protein 3	Oxidative Stress Responsive	1.098	0.044	0.224	0.113	0.009
Vim	Vimentin	Oxygen Transporters	0.920	0.165*	0.290*	0.075*	0.414*
